# A non-linear dynamical approach to belief revision in cognitive behavioral therapy

**DOI:** 10.3389/fncom.2014.00055

**Published:** 2014-05-15

**Authors:** David Kronemyer, Alexander Bystritsky

**Affiliations:** Anxiety and Related Disorders Program, David Geffen School of Medicine, Semel Institute for Neuroscience and Human Behavior, University of CaliforniaLos Angeles, CA, USA

**Keywords:** AGM theory, belief revision, cognitive behavioral therapy, cognitive restructuring, exposure/response prevention, non-linear dynamical psychiatry, systematic desensitization

## Abstract

Belief revision is the key change mechanism underlying the psychological intervention known as cognitive behavioral therapy (CBT). It both motivates and reinforces new behavior. In this review we analyze and apply a novel approach to this process based on AGM theory of belief revision, named after its proponents, Carlos Alchourrón, Peter Gärdenfors and David Makinson. AGM is a set-theoretical model. We reconceptualize it as describing a non-linear, dynamical system that occurs within a semantic space, which can be represented as a phase plane comprising all of the brain's attentional, cognitive, affective and physiological resources. Triggering events, such as anxiety-producing or depressing situations in the real world, or their imaginal equivalents, mobilize these assets so they converge on an equilibrium point. A preference function then evaluates and integrates evidentiary data associated with individual beliefs, selecting some of them and comprising them into a belief set, which is a metastable state. Belief sets evolve in time from one metastable state to another. In the phase space, this evolution creates a heteroclinic channel. AGM regulates this process and characterizes the outcome at each equilibrium point. Its objective is to define the necessary and sufficient conditions for belief revision by simultaneously minimizing the set of new beliefs that have to be adopted, and the set of old beliefs that have to be discarded or reformulated. Using AGM, belief revision can be modeled using three (and only three) fundamental syntactical operations performed on belief sets, which are expansion; revision; and contraction. Expansion is like adding a new belief without changing any old ones. Revision is like adding a new belief and changing old, inconsistent ones. Contraction is like changing an old belief without adding any new ones. We provide operationalized examples of this process in action.

Non-linear dynamical psychiatry recently has taken two different directions. The first is the granular description of neurological systems from a bottom-up, micro level, in order to characterize a cognitive phenotype such as emotion or attention (illustrative is Rabinovich et al., 2010a). The second is the functional description of psychopathology and corollary intervention strategies from a top-down, macro level, in order to characterize the course and progression of psychiatric disorders (illustrative is Bystritsky et al., 2012). Drawing on both, in this review we set forth a theory of belief revision for the intervention strategy known as cognitive behavioral therapy (CBT). CBT postulates that psychiatric disorders such as anxiety and depression are not caused by acts, transactions, events or circumstances in the real world, or by one's imaginal reconstruction of them. Rather, they result from one's attitude, orientation or outlook toward them. Persons who are anxious or depressed hold dysfunctional beliefs about themselves, others, their environment and the future. Dysfunctional beliefs are caused by an invalidating environment, deficient information-gathering practices and breakdowns in one's belief formation system (Warman et al., 2007). They often are accompanied by dysregulated emotions (Linehan, 1993). As a result, persons holding them engage in problematic or undesired behavior that is personally distressful or socially maladaptive, for example, anger, impulsivity, self-harm, self-isolation or substance abuse (“target behavior”).

Belief revision is the primary therapeutic technology underlying CBT. As we will explain, it comes in two types. The first, called “cognitive restructuring,” reformulates old beliefs and changes them into new ones. As a result, one is able to reregulate one's emotions and modify or abandon target behavior. The second results from behavioral change through a process called “systematic desensitization” or “exposure/response prevention.” It extinguishes old, conditioned target behavior and introduces new more flexible, adaptive behavior. This in turn reformulates or discards old beliefs and reregulates emotions, reinforcing the newly-learned behavior. In both cases, the new behavior then stabilizes, consolidates and strengthens the new beliefs. Both are forms of belief revision: the former, more cognitively-based than behavioral; and the latter, more behaviorally-based than cognitive. Belief revision also reduces the intensity of interoceptive alarms activated by the sympathetic nervous system when stressed, such as those characteristic of panic (Khalsa et al., 2009; Domschke et al., 2010). CBT widely is regarded as the paradigm of an empirically-supported therapy (EST) (Butler et al., 2006), which should make it particularly amenable to a cognitive science-based approach.

Our central premise is that belief revision in CBT is an integral component of a non-linear dynamical process of psychological change as conceptualized, for example, by Bystritsky et al. (2013). Anxiety and mood disorders have three essential components, which are alarms, beliefs and coping strategies (A-B-C). Alarms can be evaluated using conventional metrics such as their frequency, intensity, duration and onset. Coping strategies–a form of behavior–can be evaluated by whether they are distressful, maladaptive, or effective in down-regulating the incidence of target behavior and the intensity of correlative alarms. Beliefs are more difficult to integrate into a theory of non-linear dynamical systems. They have several unique characteristics as cognitive phenotypes, which prevent them from fitting well into the canonical model. One might not even notice one has beliefs to begin with, unless and until they are activated by environmental triggers, interoceptive sensations or undesired behavioral consequences.

Alternatively, we propose and demonstrate a set-theoretical, semantically-based approach to belief revision known as AGM theory, and show how it is the most plausible candidate to perform belief revision within a non-linear, dynamical framework. AGM is an acronym of the last names of its inventors, Alchourrón et al. (1985). It sets forth the requirements for non-delusional belief change in light of new evidence, and that one's resulting updated knowledge base must meet, in order to remain intuitively appealing (Carnota and Rodríguez, 2011, p. 2). As we discuss at §3, AGM operationalizes the cognitive component of CBT. Its objective is to define the necessary and sufficient conditions for belief revision by simultaneously minimizing the set of new beliefs that have to be adopted, and the set of old beliefs that have to be discarded or reformulated. Using AGM, belief revision can be modeled using three (and only three) fundamental syntactical operations performed on belief sets, which are expansion; revision; and contraction. Expansion is like adding a new belief without changing any old ones. Revision is like adding a new belief and changing old, inconsistent ones. Contraction is like changing an old belief without adding any new ones.

## Some relevant considerations about belief

The nature of belief and what it is to believe in something (a doxastic state) both long have been central pre-occupations of psychology and epistemology (Schwitzgebel, 2010). It is beyond the scope of this review to discuss exhaustively the voluminous literature on belief, which has accumulated relentlessly since antiquity. We will, however, briefly develop several characteristics of belief pertinent to its integration into a theory of non-linear dynamical systems, which any theory of belief revision must take into account^1^.

**Figure 1:**
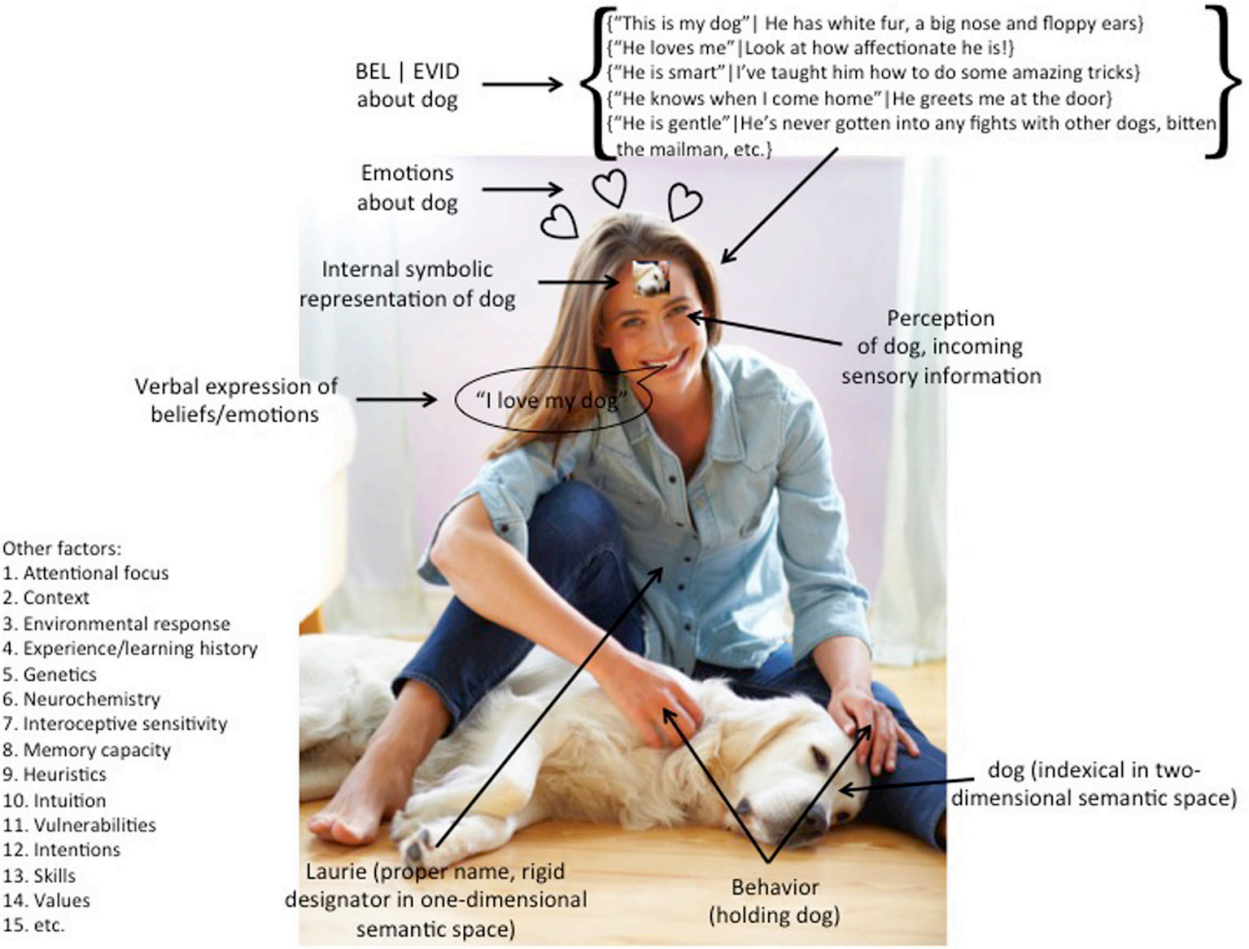
**Depiction of canonical belief model.** Photograph licensed from Getty Images

A consensus definition is that beliefs are “states of mind that have the property of being about things–things in the world, as well as abstract things, events in the past and things only imagined” (Churchland and Churchland, 2013, p. 1). Russell (1921/2005) and colleagues famously developed a theory of propositions and propositional attitudes. What beliefs are about is their substantive propositional content, i.e., (that “*x*”). Belief is an attitude, orientation or outlook toward that propositional content, i.e., BEL(“*x*”). The set of all of one's beliefs at time *t*_1_ is one's knowledge base *k*_1_. Beliefs are different than simple reference to people, places or things; informal or colloquial uses (Grice, 1975); as well as other modes of discourse such as performatives (Austin, 1962)^2^. While all of its individual elements are controversial in various respects, for our purposes, Figure 1 depicts the standard model of belief, with components including perceptual, cognitive, emotional, linguistic and behavioral processing.

### Beliefs are based on evidence

Evidence is a set of epistemological claims adduced to support a belief set. Relevant evidence enables one to devise and then test various hypotheses the belief set generates (Glymour, 1975; Hartmann and Sprenger, 2010). One is justified in believing that “*x*” to the extent one has good evidence for “*x*” (Feldman and Conee, 1985; Joyce, 2011). In the case of psychiatric disorders such as anxiety or depression, evidentiary data are things one might cite or rely on to support a contention that what one is *afraid* will occur, actually *will* occur. The feared outcome or consequence does not *actually* have to occur, rather, the evidence gives credence to the belief or prediction that it will.

From a clinical standpoint, the client is not responding to an object of fear; instead, to an internal symbolic representation of it, which (among other properties) has a compelling sense of reality. The client's behavioral expressions and coping strategies in turn are not a reaction to the feared object, but rather to the set of beliefs surrounding it, comprising the client's vision of what the feared object is, or might be. Under these circumstances, evidence is nothing more than the way things seem. One is “right to believe everything he believes as strongly as he believes it until it is rendered improbable by something else he believes” (Swinburne, 2011, p. 202). This support function often is conditional (Joyce, 2003). A conditional belief is one with the form BEL(*x*)|{EVID_1_, EVID_2_,… EVID_*n*_}, which reads “BEL(that “*x*”) assuming {EVID_1_, EVID_2_,… EVID_*n*_}” (Arlo-Costa, 2007).

In psychiatry, evidence often is clinical observations of patient behavior or patient reports of symptoms set forth in the Diagnostic and Statistical Manual (DSM-5) (American Psychiatric Association, 2013). An example of the former: BEL(“This person is depressed”) | EVID(“She has insomnia or hypersomnia nearly every day and significant weight loss when not dieting or weight gain, or decrease or increase in appetite nearly every day”). An example of the latter: BEL(“I'm depressed”) | EVID(“I have markedly diminished interest or pleasure in all, or almost all, activities most of the day, nearly every day; and I have feelings of worthlessness or excessive or inappropriate guilt nearly every day”). Evidence also can be third-person observations or patient reports of them. Example: EVID(“She always is fighting with her friends”) or EVID(“My parents always told me so”). Persons also may have corollary beliefs about their beliefs (Paulus and Stein, 2010). For example, one might BEL(“Therapy/pharmacology doesn't help”) or BEL(“I'm going to have this for the rest of my life”). They also might be reflexive, as in BEL(“I'm afraid of experiencing the symptoms of panic disorder”).

### Referential opacity

A sentence's reference is what it designates. Sentences about beliefs are referentially “opaque” in that co-designating terms are not intersubstitutable (Quine, 1953/1980). To use a famous example, Oedipus married Jocasta; Oedipus believed Jocasta was his girlfriend; Oedipus didn't know Jocasta was his mother. This reads as follows: there was a time (*t*_1_) when Oedipus believed “Jocasta was his girlfriend” (BEL_1_) given the supply of evidentiary data {EVID_1_, EVID_2_,… EVID_*n*_} then available to him. Even though true, Oedipus didn't believe at *t*_1_ “Jocasta was his mother” (BEL_2_), i.e., BEL_2_ ∉ *k*_1_. He discovered this only at *t*_2_, when (to his consternation) his knowledge base was *k*_2_.

It follows that sentences about beliefs are informative in a way that “the sum of the angles of a triangle is 180°” is not. Another famous example from Gottlob Frege: one believes the morning star rises in the east; one also believes the evening star sets in the west; one doesn't know both are the planet Venus. Even though both sentences refer to the same thing, their meanings or “senses” are different (Zalta, 2012). Failures of reference do not require one to postulate intentional conduct. They may be due to something as simple as accident or mistake (Austin, 1956/1970)^3^. The main problem with belief reports is that they rely on a client's interpretation of her subjective phenomenological experience (Dattilio et al., 2010).

### Beliefs are subjective

Referential opacity is a set-theoretical way of saying that beliefs are inherently subjective. As *homo credens*, people are infinitely capable of believing any number of different things (Shermer, 2012). One might believe in unicorns, global warming, conspiracy theories, that the sun revolves around the earth, or that they are the present King of France. It is not our intention to restrict the content of different beliefs, or the types of evidence that may be adduced to support them.

Psychiatrists and psychologists have devised numerous ways to find out *what* people believe, including observing them, testing them and asking them. In this sense, beliefs are “epistemically objective.” Implausible as it may seem, in the near future, it might even be possible to read a person's mind using neurotechnologies such as fMRI (Harris et al., 2008; Poldrack et al., 2011); neuropsychiatric phenomics (Bilder et al., 2009a,b); connectionist-type principles (Sporns et al., 2005); or interactionist-type principles (Stumpf et al., 2008)^4^.

One of the perennial issues in cognitive science is whether these methods ever will be sufficient to account for belief's phenomenological texture. There is something unsatisfying about the neuromaterialistic/neurodeterministic program of extracting the substantive propositional content of a belief from neurological events. The reason why is because beliefs are underdetermined neurophysiologically; a single neurological state potentially could give rise to any number of different beliefs (they are “multiply realizable,” (Levine, 1983, 1999); there is an “explanatory gap” between the two, Davidson, 1970, 1974). Further, they only can be held by the person who believes them. In this sense they are “ontologically subjective,” as features or ascriptive predicates attributable only to that person (Dehaene, 2014, p. 9; Searle, 1995, pp. 7–9)^5^. From a clinical standpoint, there is no such thing as a standardized set of beliefs. Any approach to psychometric assessment that attempts to construct a taxonomy of typical beliefs, whether normative or pathological, most likely will not be successful, because beliefs fundamentally are distinctive, unique and personal. The clinician and the client must become co-investigators to identify them and the evidence ostensibly supporting them.

### Beliefs are mediated and moderated

Beliefs are mediated and moderated by any number of different factors such as background, upbringing, life experiences, information processing strategies, temperament, attributional style, other beliefs, context, culture, motivation, and the presence of environmental cues and situational primes (Hope et al., 2010). They may be teleological or subject to confirmation bias. People deploy a variety of heuristic reasoning strategies to arrive at the beliefs they hold, including hypothesis formation, generalization and anomaly resolution. Reasoning has a rational basis rooted in probabilistic approaches to problem-solving (Kahneman and Tversky, 1979; Tversky and Kahneman, 1983; Oaksford and Chater, 2007). These strategies have evolved over time to facilitate our ability to make decisions in situations with incomplete information as to potential outcomes (Kahneman et al., 1982; Shafer and Tversky, 1985; Kahneman, 2003; Michalewicz and Fogel, 2004). They include everything from educated guesses to intuitive judgments and common sense. Induction is an important aspect of human reasoning (Heit and Rotello, 2010; Johnson-Laird, 2010), as are techniques to evaluate the evidence in support of individual beliefs such as Bayesian reasoning and Dempster-Shafer theory (Curley, 2007; Zhao and Osherson, 2010; Zhao et al., 2012). There also is a complex relationship between cognition and emotion (§2.1.4, below; Pessoa, 2008, 2014). Beliefs are thought; emotions are felt. Just as one can have beliefs about one's emotions, so does one's emotional state affects one's belief-generating system. As with the subjective nature of beliefs (§1.3, above), while all of these are controversial in various respects, it is not our intention to restrict the nature, scope and extent of potential belief influencers.

### Conditions of satisfaction

A proposition has the property that it is true or false in the real world (McGrath, 2012). Beliefs, on the other hand, have conditions of satisfaction–what happens when things are the way one believes them to be. BEL(“It's raining”) is satisfied if in fact it is raining. Under those circumstances, we say the belief is “true.” Beliefs have a “mind-to-world” direction of fit, in that the belief corresponds, to some extent, with reality (Searle, 1983).

### Psychopathology disrupts the entire belief template

One of the best ways to consider belief as a psychological construct is to examine counterfactual cases (Langdon and Connaughton, 2013). Persons who are anxious or depressed have beliefs that are dysfunctional and experienced as negative and invalidating (Bernstein et al., 2010, 2013). Example: BEL(“If I try to do this, I'm going to fail”).

The main problem with dysfunctional beliefs is they cannot be assigned a truth value, as in BEL (“The cat is on the mat” | There is a creature of the genus and species *felis catus* lying prone upon a rectangle of flooring material). Rather, one *thinks* conditions of satisfaction have been met, or thinks *others* think they have, when in fact they have not. Example: BEL(“I'm a terrible person”) does not imply one in fact is a terrible person (under some plausible consensus definition of what that means), or that others think so. Initially, negatively-valenced beliefs arise from misinterpretation of exteroceptive and interoceptive evidence and from information processing deficits (Paulus and Stein, 2010; Boden et al., 2012). Misevaluation of conditions of satisfaction then causes one to misjudge the evidence supporting the feared outcomes (“cost biases”) (Nelson et al., 2010a,b).

Normatively, we are inclined to impose certain minimum requirements on a set of beliefs in order to maximize the likelihood there will be a match between beliefs and conditions of satisfaction. These include conformity, conditioning and coherence (Howson, 2009).

### Conformity

Conformity disregards the substantive propositional content (“*x*”) of BEL(“*x*”) and requires only that one not endorse (“-*x*”) simultaneously. Actual human reasoning might not be quite that simple. Research shows that people deal with inconsistencies not by attempting to refute one of the premises, but rather by trying to explain their origins, which has the side effect of revising their beliefs (Khemlani and Johnson-Laird, 2011).

### Conditioning

Conditioning means that one should hold BEL(“*x*”) only for so long as {EVID_1_, EVID_2_,… EVID_*n*_} support (*x*) and that one must update (*x*) in light of new, incoming EVID. Such an update may involve modifications to the belief's conditions of satisfaction. Acquiring, maintaining and using new evidence in order to revise and update beliefs is a crucial human survival strategy (Patterson and Barbey, 2013). When incorrect or obsolete, conceptual knowledge must be repaired by integrating and explaining new material (Friedman and Forbus, 2011).

### Coherence

Coherence means that only tautological falsehoods qualify for a probability assignment of *p*(*x* = 0) and only tautological truths qualify for *p*(*x* = 1). Thus one should not assign *p*(BEL) = 0 to (BEL = “the sum of the angles of a triangle is 180°”), §1.2, above. Rather, one should assign it *p*(BEL) = 1.

Although they seem sensible, these axioms often do not apply to psychopathological states, because cognitive processing systems are impaired and emotion processing systems are dysregulated. Persons holding dysfunctional beliefs also may not be able to reason normatively. For example, they may disbelieve a set of propositions (e.g., evolution, global warming), which (most) everybody else believes (Perring, 2010). They may be indifferent to antecedent beliefs and stored knowledge; misunderstand inferential relationships; prioritize anomalous perceptual experiences; and lack a coherent theory of mind (Davies and Coltheart, 2000). It also makes sense to think of sentences expressing the ideations of persons with psychiatric disorders (§1.2, above) as ultra-opaque, thus even less amenable to substitutability of identity.

Their ability to evaluate evidence also may be impaired. Normatively, one relies on evidence to support a belief that what one *thinks* will occur, actually *does* occur. The evidence does not contradict, and in fact supports, the belief. In problematic cases, though, one does not have to believe a feared outcome or consequence actually *will* occur. Rather, all one has to believe is that the evidence supports the *belief* that it will, regardless of whether it happens or not (Joyce, 2011; §1.1, above). In such cases, the evidence supporting the belief is misaligned with reality (Warman et al., 2007; Möller, 2012). Clearly this is a slippery slope. If people can believe whatever they want, then what's to stop them, particularly if they have a mental disorder?

### Subjective probability theory

There are two modern epistemic interpretations of probability, which are logicism and subjectivism (Galavotti, 2011). Logicism contends that probability is a person-independent, normative relationship between real-world facts or events. Subjectivism is the theory that probability is one's degrees of belief (Hájek, 2011). Under the logicist interpretation, a tautological statement (such as *A* → *B*; *A*; ∴ *B*) is certain regardless of what people may think about it. Its probability *p* within a sample space Ω is 1 and in principle a large number of other beliefs can be incorporated within Ω so long as they are complementary (§1.6.3, above). Under the subjectivist interpretation, different persons can believe whatever they want and assign their beliefs different *p*-values, even given the same evidence, permitting wide intersubjective belief variation.

Subjectivism almost certainly is true when considering a person's individual beliefs (§1.3, above). It breaks down, however, when considering a set comprising different beliefs, all held by the same person. This surely is normative. It would be odd for a person only to have one belief. Most people probably hold tens of thousands, perhaps hundreds of thousands, of beliefs, and their knowledge base most likely expands over time (Ohlsson, 2011, p. 293). The problem is not about subjectivism. Rather, it is about probability. Probability assessments do not occur on an interval scale, making it impossible to combine them or determine something analogous to their “mean” probability function using a linear pooling methodology (Wallsten et al., 1997)^6^. Beliefs comprising belief sets are interdependent, not independent. As a result, they cannot be evaluated using a differential equation or structural equation modeling approach. A differential equation approach will not work, because one cannot parameterize the values of the variables in order to create a belief change trajectory or phase portrait within a vector field. A structural equation modeling approach will not work, because one needs dimensionality reduction. For example, if one holds 13 separate beliefs, the binominal coefficient is 715. Their interaction effects are 13! (13 factorial), or 6,227,020,800. Beliefs simply cannot be converted into numbers. They are not variables with values. Consequently, there must be some other way to fit beliefs into a non-linear dynamical model.

### Beliefs have semantic, propositional content

The solution is that beliefs have semantic, propositional content. Semantic content need not be expressed in complete sentences or even phrases. It can be concepts that either are the semantic content or that combine to form it (Laurence and Margolis, 2012). Beliefs are just such a conceptual state. Unlike variables populated by values, they must be elicited using a natural language and then comprised into sets at various stages of the belief generating process (*t*_1_, *t*_2_,… *t*_*n*_). One selects beliefs and includes them as members of belief sets by promoting or prioritizing them ahead of others, based on one's credences in the evidence supporting them, or levels of confidence in their conditions of satisfaction (§1.5, above; Makinson, 2009; Dietrich and List, 2013). Credences are situated along a continuum ranging from complete certainty of falsehood (does not meet perceived conditions of satisfaction) to complete certainty of truth (meets perceived conditions of satisfaction), depending on the evidence (Joyce, 2009).

#### Preference functions

Individual beliefs are organized into sets by preference or ranking functions (γ), which assess the occurrence or persistence of the belief (Spohn, 2009). In order to assign a preference function, one must adopt a theory of utility to determine what counts as a desirable (utility-maximizing) action; establish degrees of belief; rank preferences; and determine what evidence counts as confirming what beliefs (Johnson-Laird, 2010, 2013; Meacham and Weisberg, 2011). The higher a belief's preference function, the more likely it is to provide a basis for behavior (Segerberg et al., 2009)^7^. Following this compilation process, different belief sets then can be evaluated in order to determine the nature, scope and extent of belief revision, most likely by a human skilled in use of the language in which the beliefs are expressed^8^. It is likely that different beliefs impose contrasting and disparate semantic burdens, based on factors such as prevalence, complexity, and the number of inferences involved.

#### Semantic encoding

An example of a technique that has been devised to elicit beliefs is the articulated thoughts in simulated situations (ATSS) think-aloud paradigm, initially developed by Davison et al. (Zanov and Davison, 2010). Computational semantics attempts to model key features of natural language processes such as word meaning, sentence meaning, pragmatic usage and background knowledge (Stone, 2014). Recent initiatives include WordNet (Princeton University, 2010); latent semantic analysis (LSA) (University of Colorado Boulder, 1998); and SNePS (SNePS. Research Group., 2013). WordNet is a lexical database that groups words into sets of distinct cognitive concepts. LSA evaluates word similarity by similarity of context of use. SNePS is a natural language knowledge representation and reasoning system. A SNePS sub-routine models belief revision to maintain conformity, conditioning and coherence (§1.6.1, §1.6.2, §1.6.3, above). It too requires both individual beliefs and their relationships to be semantically encoded. One of the research priorities of several of today's most prominent internet companies is to develop algorithms for natural language recognition. Apple acquired Siri in April 2010 (Wortham, 2010); Facebook announced Graph Search in January 2013 (Sengupta, 2013); Google announced Hummingbird in September 2013 (Miller, 2013); Yahoo announced SkyPhrase in December 2013 (Goel, 2013); and in February 2014, Wolfram released software intended to answer natural language queries with real-world information as a kind of “computational knowledge-engine” potentially demonstrating a form of “machine intelligence” (Lecher, 2014). One of the main challenges of these initiatives will be to capture the numerous shades and nuances of meanings used by fluent language speakers–the senses of words, in Fregean terms (§1.2, above).

#### Semantic entailment

Closely related are problems of semantic entailment, that is, when a phrase or sentence commits one to other associated concepts. A classic example: “Socrates lived in Greece” should be inferred from “Socrates lived in Athens.” Words are organized into “semantic/associative neighborhoods within a larger network of words and links that bind the network together” (Nelson et al., 2013, p. 797); Schroeter (2012) characterizes it as a two-dimensional semantic space comprising rules for assigning values to words and sentences. Specifying exactly what these neighborhoods and networks are is challenging, because (as with semantic encoding, §1.8.2, above) it depends on acquiring paraphrases, lexical semantic relationships, and inferences in contexts such as question answering, information extraction and summarization–similar to the usages employed by a natural language speaker (Dagan et al., 2009).

### Beliefs do not exist in isolation

As semantic entailment illustrates, beliefs are components of complex domains, knowledge sets and networks (Davidson, 1994/2005). The limits of certitude on the one hand and psychopathology on the other allow for a wide variety of different {BEL | EVID} (Huber, 2009). One has an extensive set of unspecific background beliefs, which are culturally sensitive and context-dependent. They are “encoded in our linguistic formulation of the problem” (Weisberg, 2011, p. 507). Activities such as data selection, acquisition and learning require constant revision to one's knowledge base. Belief formation is subject to the overwhelming intervention of human experience, chance events and real-world constraints (Oaksford and Chater, 2007).

Quine and Ullian (1978) refer to this as a “web of belief”–“The totality of our so-called knowledge or beliefs, from the most casual matters of geography and history to the profoundest laws of atomic physics or even of pure mathematics and logic, is a man-made fabric which impinges on experience only along the edges” (Quine, 1953/1980, p. 42). Another way to look at beliefs is how they fit into what Searle (1995) calls the “background”–“all of those abilities, capacities, dispositions, ways of doing things and general know-how that enable us to carry out our intentions and apply our intentional states generally” (Searle, 2010, p. 31); or, the “foundational, non-representational non-rule-governed, dispositional structure of everyday understanding that underpins both our perception and our reasoning” (Rhodes and Gipps, 2008, p. 295).

### Dynamics of natural language formation

Another important factor involved in belief semantics is the dynamics of natural language formation. Any language must have certain minimal constructs and features. These include generativity (one can create an indefinite number of new sentences from its component elements); discreteness (semantic elements, such as words, retain their identity, even in different syntactical contexts); compositionality (smaller language units, such as words, can be combined to form more complex ones, such as sentences); predictability; and recursion (phrases can be embedded within phrases to create new sentences) (Hauser et al., 2002; Studdert-Kennedy, 2005; Searle, 2007). Noam Chomsky famously theorized there was a universal human linguistic structure, which he called “generative grammar” (Chomsky, 1955, 1965). For Chomsky, syntax was the essential component of language, as opposed to semantics (meaning and reference) and pragmatics (how language actually is used) (Chomsky, 1977)^9^.

### Language and mind

It is beyond the scope of this review to investigate the complex relationships between language and mind (for a current overview, see Gleitman and Papafragou, 2012, 2013). Issues include criticism of Chomsky's views; whether logical variables represent the propositional contents of mental states and that cognition consists in manipulating them, a view most closely associated with Jerry Fodor (1975); criticism of Fodor's views; the linguistic relativity hypothesis (Swoyer, 2003); whether one can observe thoughts or emotions without labeling them (Linehan, 1993); or whether simply changing the way one labels them is effective to initiate cognitive/affective/behavioral change (Lieberman et al., 2007; Hayes et al., 2012). Our concern is not just a matter of choosing new words to describe beliefs, but rather reformulating beliefs, which then are expressed using words. At a minimum, we are in accord with Davidson (1975), who holds that belief is central to thought and that to have a belief requires the ability to express it using words^10^.

The substantive propositional content of an individual belief is interesting and important, particularly for determining just which dysfunctional beliefs typically align with different types of psychopathology. We are more interested, though, in the relationship of an individual belief to the other constituents of the belief set of which the individual belief is a member, and how that set's membership changes or is reformulated between *t*_1_ and *t*_*n*_. Belief revision does not involve alteration or replacement of that which the belief is about, i.e., the “*x*” in BEL(that “*x*”). It is not a form of reality modification. Rather, the focus of change is belief considered as a propositional attitude (§1, above). The nature, scope and extent of belief revision only can be evaluated by inspecting modifications to the semantics of sets of {BEL | EVID} at *k*_1_ and *k*_*n*_.

### Integrating belief into a non-linear dynamical system

**Figure 2:**
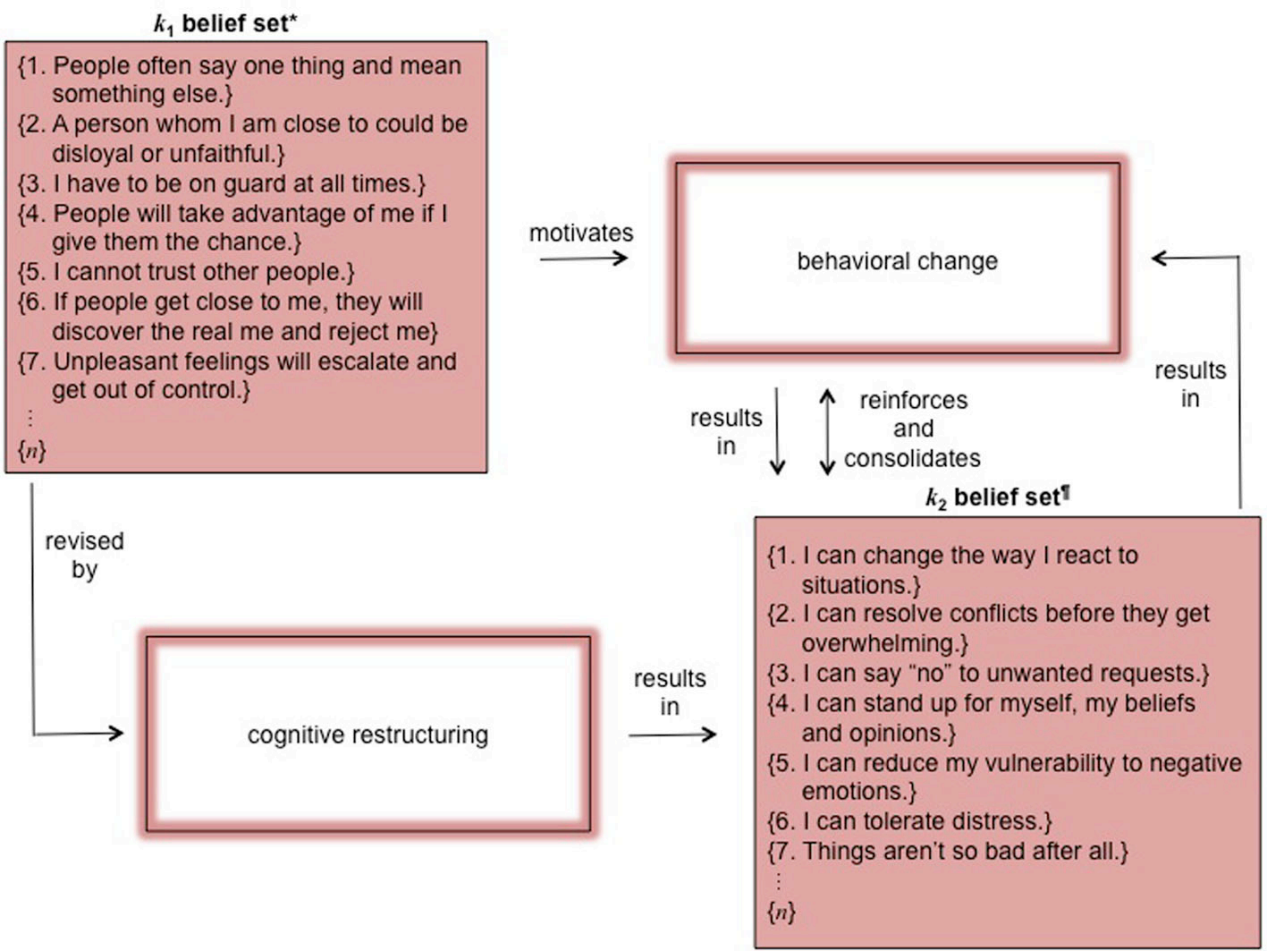
**Schematic of non-linear dynamical belief revision processes in CBT.** ^*^Adapted from Bahr et al., 2008. ^¶^Adapted from Linehan, 1993. *k*_1_ is one's knowledge base at time *t*_1_; *k*_2_, at *t*_2_; this example uses beliefs characteristic for a person presenting with symptoms consistent with a diagnosis of borderline personality disorder.

Given these complex conditions, how can belief revision using CBT be integrated into a theory of non-linear, dynamical systems? As set forth at our Introduction, above, belief revision essentially involves two separate pathways: one through cognition, the other through behavior. CBT straightforwardly uses interventions directed toward both. The first, cognitive restructuring, requires belief revision in order to initiate behavioral change. The second, exposure/response prevention, requires behavioral change in order to initiate belief revision. Both cognitive restructuring and exposure/response prevention are mechanisms of belief revision from *k*_1_ to *k*_2_ (*k*_1_ Δ*k*_2_). Figure 2 illustrates their respective critical paths for a client presenting with borderline personality disorder, DSM-5 §301.83.

### Cognitive restructuring

Cognitive restructuring is the therapeutic technology underlying the “cognitive” component of CBT (Spiegler and Guevremont, 2009). It contends that belief revision is the active ingredient motivating behavioral change: if belief set *k*_1_ at time *t*_1_ is modified to belief set *k*_*n*_ at time *t*_*n*_, then more adaptive behavior will follow (Leahy, 2001, p. 23). Cognitive restructuring erodes dysfunctional beliefs through several steps: (1) identify them; (2) marshal disconfirming evidence against them; (3) deconstruct them by challenging and refuting them; (4) replace them with alternative, more functional beliefs; and then (5) conduct behavioral experiments to see how the environment responds (Huppert, 2009; McMillan and Lee, 2010; Morina et al., 2011). Examples of cognitive-oriented interventions include decatastrophizing, disputing the evidence, detecting logical errors, chain analysis, situational analysis, etc. (Leahy and Rego, 2012).

Clinical interventions look something like these: If one is afraid of snakes, that belief can be challenged through a series of counter-examples. A herpetologist might be concerned with the snake's various anatomical features. A veterinarian might be concerned with its health. A herpetoculturist might be concerned with its taxonomy. Some people have them as pets, or pose with them for photographs, or perform with them in theatrical productions. Each of these persons has a different, proactive mental stance toward things that are (or that appear to be) snakes, none of which are threatening. Or, if a person with lived experience concedes suicidal ideations or reports parasuicidal target behavior, then one way to interrupt her might be to evaluate the evidence and establish the active ingredients of a life worth living: “We have no reliable information that persons who are dead have a better quality of life than persons who are alive. If you're dead, then therapy won't work and you won't be able to get better.”

It follows that in order to recalibrate one's belief-generating system, one must modify one's credences in the evidence supporting the pathological belief. The first step in cognitive restructuring is to elicit BEL(*x*). Then, for example, BEL^⊖^(“I'm afraid of *x*”) at *t*_1_ might get cognitively restructured into something like BEL^⊕^ (“There've been times when I've encountered *x* and it wasn't so bad”) at *t*_2_. Positive belief attributions (BEL^⊕^) supplant negative ones (BEL^⊖^). Following cognitive restructuring, one then searches for discrepant evidence to confirm BEL^⊕^ and disconfirm BEL^⊖^, giving one a good reason to reformulate one's behavioral repertoire (Garland et al., 2010; Morina et al., 2011; Lightsey et al., 2012). Like belief, fear simply is another propositional attitude, i.e., {fear(*x*) | EVID}. Once one has accumulated enough relevant evidence, the choice clearly is framed: spend a significant portion of one's time entrained to the feared outcome, vs. the likelihood it actually will occur (i.e., conditions of satisfaction will be met, §1.5, above). From an assessment standpoint, this likely would require one to have good metacognitive awareness, that is, the ability to reflect upon, understand and control their learning (Schraw and Dennison, 1994) in order to be able to identify and articulate their beliefs. A related concept from attachment theory is that of reflective functioning, that is, the ability to observe and describe one's own mental state (Fonagy et al., 1991).

Cognitive restructuring presents several issues:

1. It is difficult to challenge entrenched beliefs, even when they result in target behavior. Although maladaptive, to some extent they relieve immediate personal distress. Over time they are reinforced and become a conditioned response to the circumstances triggering them, which consolidate around their utility and effectiveness (Hartley and Phelps, 2012).
  Example: aerophobia (fear of flying). In effect one has become fear-conditioned: the unconditioned stimulus (flying) initially provokes anxiety (unconditioned response), then becomes paired or associated with other typically-innocuous contexts or situations extrapolated from or analogized to the original one (such as acrophobia, fear of heights, the conditioned stimulus) (Samanez-Larkin et al., 2008). The resulting thought-pathways become ingrained with experience as they are reinforced by sufficient confirming evidence that maintains the associated beliefs until they become conditioned, learned responses (Tryon and McKay, 2009). One keeps doing the same thing over and over again because one is afraid of the perceived consequences of doing anything else.
2. Cognitive restructuring readily can morph into a form of escape/avoidance, if misapplied, because it feeds into intellectualization rather than the emotional, felt experience of a genuinely feared outcome. From a clinical perspective, too much thinking can become therapy-interfering, because one might approach the feared outcome as a puzzle to be solved. If this happens, then cognitive restructuring might backfire and one's tolerance of the feared outcome deteriorates even further. Feelings and thoughts both are in continuous competition for the same cognitive resources.
3. Because it involves a series of complex mental events, cognitive restructuring may be too complicated for many persons, especially those presenting with delusional features or severely dysregulated emotions (§4, below). They barely may be able to tolerate their dysfunctional beliefs, much less generate new ones. Persons with body dysmorphic disorder (BDD), for example, have a granular information processing style so they recall selective details of their appearance, rather than larger organizational design features (Feusner et al., 2007). This makes it difficult for them to generalize from a specific exposure addressing a particular feared outcome to more global cognitive change. While one might become inoculated or desensitized to a particular trigger, establishing it also applies in other contexts requires deducing there is a more pervasive relationship between them–which is a cognitive process. In effect one must blunt the impulse toward fractalization.

If one adopts the wrong cognitive hypothesis, then it will be ineffective to revise the associated belief set. In order to be successful, cognitive restructuring must correctly identify the ultimate fear: “I'll lose control,” “I'll be judged,” “I'll be embarrassed and humiliated,” “I'm going to die,” etc. If one is afraid of physiological symptoms such as those characteristic of panic, then the question should be, what happens next? For example, if a client presents with symptoms consistent with a diagnosis of social anxiety disorder (SAD), such as vasodilation (blushing), then the consequence might be that “people think I'm an idiot.” If people think one's an idiot, then the next consequence might be “I'll be rejected and abandoned.” If one's rejected and abandoned, then the next consequence might be “I'll lose my job and my relationships,” etc. If the terminal fear is not adequately specified, then target behavior actually might increase over baseline, because rather than contending with dysfunctional beliefs, one just has animated or enlivened them. The reason why is because one *thinks* one has handled the problem, but one really hasn't (§1.6, above). One just has deferred dealing with it. As a result, further triggers will continue to recruit and redeploy cognitive, affective and physiological assets to support it (Smits et al., 2008; Olthuis et al., 2012).

4. Cognitive restructuring essentially is a process of “out with the old, in with the new” using interventions such as those described at §2.1, above (Leahy and Rego, 2012). Because CBT regards dysfunctional beliefs as distortions or errors in thinking, such a challenge might be experienced as emotionally invalidating (Leahy, 2001, p. 58; Linehan, 1993, p. 92). Familiar (and to some extent serviceable) beliefs may be revealed as unrealistic, mistaken, distorted, or even irrational. As a result, subsequent behavior might just exchange one cognitive/affective state (e.g., anxiety) for another (e.g., “I'm deficient” or “I'm defective”). In this respect, dialectical behavior therapy (DBT) augments CBT case conceptualization. It emphasizes emotional validation in addition to cognitive restructuring. It is not enough to focus only on beliefs and behavior, because emotions (and their associated interoceptive sensations) also are an integral component of the same equation. In fact, if anything, in a contest between emotions and cognitions, emotions most likely will win out, because they are more fundamental and, in a sense, primordial (LeDoux, 1996; Damasio, 1999; Afraimovich et al., 2011; Frazzetto, 2013). A recent study by Moser et al. (2014) concluded that positively reinterpreting negative emotional experiences (such as those associated with fearful outcomes) is one of belief revision's key mechanisms, with well-defined neurological correlates. The equation *should* read: {dysfunctional beliefs} + {emotional dysregulation} = {target behavior}^11^.
5. CBT uses phrases such as “downward arrow technique” (Persons et al., 2006) and “chain analysis” (Lynch et al., 2006) as metaphors for complex cognitive processes, without considering their component elements. This leaves beliefs in a kind of mysterious “black box”–something everyone knows must be addressed, but without unpacking their underlying logic and structure. What CBT lacks (and what we offer) is a theory of belief revision–which beliefs get changed, why those instead of others, and what the constraints are.
6. Cognitive therapy is a means to behavioral change, not an end in and of itself. During cognitive restructuring, one develops hypotheses that exposure/response prevention either will falsify or prove. For example, if a person with SAD undergoes cognitive therapy and concludes, “Well, I guess it's not so bad if I speak up at meetings,” but then never does so, cognitive restructuring will not have been effective.

### Exposure/response prevention

CBT's second critical path is behavioral intervention based around the concept of progressive desensitization-exposure/response prevention to a feared outcome, rather than escape/avoidance of it. It proposes that the main driver for therapeutic change is behavior, not cognition. It assumes that it is difficult for cognition alone to motivate new behavior; that one of the main reasons why persons engage in target behavior is to attempt to induce their environment to respond; that when reinforcement contingencies are altered, behavioral modification follows; and that psychological change occurs as a result. Instead of being the driving force motivating behavioral change, cognition brings up the rear. This dichotomy is similar to that between thought and action, or thinking vs. doing.

Using this approach, the first question always must be “how did the behavior get to be the way that it is.” Often this can be explained using classical and operant conditioning paradigms. Sometimes people enact coping strategies to prevent something bad from happening; occasionally, it may even be pleasurable. If, however, actions have *not* had effects, then it is necessary to supply them in order to consequate that behavior. The next step is to unpair or decouple a conditioned stimulus from an unconditioned one, or to extinguish target behavior that previously has been reinforced (and the entire cycle giving rise to it), by establishing prospective environmental contingencies; acquiring skills; enacting new behavior; and then evaluating evidence as to how the environment responds (Spiegler and Guevremont, 2009). At each stage, behavioral markers demonstrate that the feared outcome did not occur.

Target behavior typically is a form of escape/avoidance. It may be accommodating and protective in the short term, because it reduces the threat posed by dysfunctional beliefs (§2.1.1, above; Hofer, 2010). However, it is ineffective over the long term, as novel and even more threatening stimuli arise in the world and present for interpretation and action (Roemer et al., 2002; Carter et al., 2008; Lee et al., 2010). It does not affect one's pre-existing vulnerabilities and the environmental affordances that trigger or activate them. It does not down-regulate dysfunctional beliefs or dysregulated emotions. Instead, by impeding assimilation of accurate information, it maintains judgmental biases, emotional vulnerability and alarm sensitivity–a kind of “contrast avoidance” (Taylor and Alden, 2010; Newman and Llera, 2011, p. 226).

Adaptive new behavior, on the other hand, is generated by stepwise exposure followed by systematic desensitization or response prevention. Initially this is a “fragile behavioral state” and can be recovered “spontaneously or subsequent to environment influences, such as context changes or stress” (Herry et al., 2010, p. 599). As one confronts the feared stimulus, the fear becomes extinguished through a reverse inhibitory learning process, allowing for more flexible control of conditioned response by forming a consolidated extinction memory. With continued or reinitiated exposure, post-behavior cognitions consolidate and become further refined, dampening responsiveness in the brain's fear-sensitive network (Hauner et al., 2012; Trouche et al., 2013). Similar to cognitive restructuring (§2.1.3, above), in order to be an effective intervention, exposure/response prevention must be autogenic, i.e., personalized more or less exactly to falsifying or validating a specific feared outcome–the one that matters the most.

Example: if one is afraid of heights and things that move quickly, then an escape/avoidance strategy would be not to engage with them. An exposure/response prevention strategy, on the other hand, would be to take opposite action by (say) going on a series of roller-coaster rides at an amusement park, starting with those that are small and innocuous but then building up over the course of a day to those that are taller and faster. At each step one take's stock of one's mental condition, notices that one still is alive and breathing, thereby habituating or acclimating oneself to more challenging stimuli, resulting in cognitive change. Example: if one is afraid of driving on the freeway, then an escape/avoidance strategy would be to take surface streets. What happens, though, if the surface streets all are blocked and the only way to get to one's destination is by taking the freeway? The escape/avoidance strategy no longer works. A more adaptive exposure/response prevention strategy would be to progressively expose oneself to driving on the freeway by (say) traveling from one on-ramp to one off-ramp at a time, then gradually building this up to two, then three, etc. Example: rather than engaging in a difficult and potentially futile process of weighing pros and cons in order to motivate herself not to drink alcohol, a person with substance over-use issues alters her behavioral regimen not to drive by liquor stores and restructures her social network to exclude those persons maintaining it.

Behavior modification is powerful. Some theorists contend that in a contest between beliefs and behavior (i.e., cognitive restructuring versus exposure/response prevention followed by belief consolidation), behavior always will win; see e.g., Gipps (2013) and Longmore and Worrell (2007). Historically, committed behaviorists denied one has beliefs to begin with; rather, one only is disposed to respond to stimuli (Pavlov, 1927/2003; Skinner, 1947; Ryle, 1949/2009). Today, along similar lines, eliminative materialists such as Churchland and Churchland (1998) and Dennett (1992) deny beliefs are anything more than folk-psychological explanations (this phrase is intended to be mildly derisive) of complex neurological events (Bickle et al., 2010). The weakness of this formulation is what originally lead to the cognitive revolution, as exemplified, for example, by Chomsky's (1959) critique of Skinner's (1957/1991) *Verbal Behavior*. Behavior does not, however, occur in a vacuum. There must be some threshold level of belief revision in order to stimulate it, most likely based on the salience of an initial belief or belief set, its relevance to current goals, or its resonance with a particular feature of the environment. In principle this should be similar to the way that intention redirects attention from the default mode network to some other neural construct or constructs (Buckner et al., 2008; Rabinovich et al., 2012a). Attention focuses intentional orientedness, causing heightened self-monitoring, resulting in greater interoceptive sensitivity (Simmons et al., 2006; Woody and Nosen, 2009), one of the main precursors to belief change.

Thereafter, the role of cognition primarily is to consolidate revised beliefs and build behavioral insight. Beliefs are conjectures or predictions about conditions of satisfaction and the evidence supporting them. The only way to accumulate evidence is by enacting behavioral experiments and seeing what happens. From a clinical standpoint, the client can assume the role of an anthropologist, investigating the behavior of a strange tribe, of which she also happens to be a member. If there is insufficient evidence to support a belief, or the evidence disconfirms it, then there is no particular reason why it should be retained as a component element of a belief set. Discrepant evidence creates “expectation violations” (disconfirms pathogenic beliefs), modifying behavioral vectors previously directed toward averting feared outcomes, thereby raising the cognitive accessibility of alternative and more flexible belief formulations. In many instances, the cognitive objective is not to eradicate fear, but rather to tolerate ambiguity. Using a variation of the Rescorla and Wagner (1972) model, Craske et al. (2012) recently advocated that while it may become semi-perturbed, the pairing or coupling between the conditioned stimulus and the unconditioned stimulus never really is eradicated. Instead, it is inhibited or attenuated. It follows that variability in fear level, or reintroducing elements of the unconditioned stimulus concurrently with the conditioned stimulus during exposure, is more likely to create a durable learning experience. Doing so *maximally* violates expectations, eliciting more improvisational and extemporaneous behavior, thereby promoting belief revision (Kircanski et al., 2012). The goal is not so much extinction (from a behavioral standpoint) as it is acceptance (from a cognitive standpoint)—which is a completely different skill. As the Viennese novelist (and, in retrospect, proto-ACT theorist) Robert Musil (1930-43) declared: “one must live with uncertainty, yet not be caught in hesitation.”

Cognition also extrapolates or pluralizes revised beliefs to analogous contexts. When one masters a skill in a certain domain, that mastery experience carries over to others. Only the target behavior will be affected without generalization effects. While this may be acceptable insofar as it goes, especially in refractory cases, exposure/response prevention will have limited success unless it also addresses adjacent beliefs (Arntz, 2002; Bryant et al., 2003). To continue with the example from §2.1.6, above, if a person with SAD starts mindlessly speaking up at meetings, that will not in and of itself change cognition. It simply is a form of unregulated exposure/response prevention. It may even become a form of escape/avoidance if she engages in it unthinkingly in order to avoid cognitive dissonance, a necessary precursor to extinction. The more that target behavior is effective as a form of escape/avoidance, the more difficult it will be to create a counteracting exposure/response prevention, precipitating belief revision. Reciprocally, some persons who hold severely dysfunctional beliefs or who are considerably emotionally dysregulated may lack the cognitive capacity to perform generalization operations (§4, below). In such cases, target behavior must be specified even more precisely, otherwise it will not be extinguished, or some other undesired behavior will be reinforced instead.

### Automatic negative thoughts, intermediate beliefs, core beliefs

How do cognitive restructuring and exposure/response prevention integrate with the epistemology of CBT? Received Beck-Ellis theory (Ellis, 1994; Beck, 2011) holds that doxastic agents have a hierarchy of automatic thoughts, intermediate beliefs and core beliefs. There now are several dozen recognized schools of CBT, all of which trace their provenance back to Beck and Ellis (Emmelkamp et al., 2010).

#### Automatic thoughts

For Beck (2011), automatic thoughts are an undercurrent of cognitions and self-talk, subject to articulation on query or in response to an analogous simulation (Zanov and Davison, 2010). They rarely are conscious in the sense of a state one is aware of, however they typically are accessible and available to other cognitive processes (van Gulick, 2004).

#### Intermediate beliefs

Automatic thoughts are linked to core beliefs by intermediate beliefs. Beck (2011) assumes the role played by intermediate beliefs is unproblematic (p. 205), however they can be difficult to formulate and it is not clear anybody ever has held an intermediate belief. In principle they should be rules or assumptions in the form of conditional if-then statements such as: “If I (engage in rigid behavioral coping pattern), then (I'll be insulated from a core belief I'll experience as aversive)” or “Unless I (engage in rigid behavioral coping pattern), then (I'll be exposed to a core belief I'll experience as aversive).” For example, if one unexpectedly is running late for work because the bus is running late, intermediate beliefs might be: “If I'm always on time for meetings, then I'm not inadequate” (or, “Unless I'm always on time for meetings, then I'm inadequate”). They should not, however, be idiographic. Thus, “If I'm on time for meetings, then I'll do well at work” is not a proper formulation of an intermediate belief. Rather, it is more of an expression of a particular coping style, connecting to an individual instance of behavior, not a pattern of behavior. Nor should intermediate beliefs be depersonalized. Thus, “People who frequently are late for meetings typically end up losing their jobs” also is not a proper formulation of an intermediate belief, because the outcome does not tie to a more generalizable core belief.

#### Core beliefs

A core belief is not an actual thought in an epistemological sense. E.g., if the automatic thought is “I'm running out of money,” then the associated core belief might be, “One needs a lot of money in order to be safe,” even though one never actually thinks that particular core belief. Uncovering it is cognitive restructuring's *raison d'être*. It is tempting to think of a core belief as an implicit conclusion derived from the application of a rule (an intermediate belief) to a premise (an automatic thought). All three are components of an information processing system (Beck, 2011, p. 33) or a way for people to “organize their experience in a coherent way in order to function adaptively” (Beck, 2011, p. 35).

Still, it is not clear what comprises a set of core beliefs. Is it just a single belief, or a set of multiple, interdependent beliefs? Although they acknowledge the possibility that there are many of them, all of the Beck-Ellis examples treat beliefs as singletons rather than as elements of belief sets. It seems implausible that individual beliefs, regardless of how entrenched, proximately cause (or explain) a complex phenomenon such as human behavior. It seems more likely that human behavior is the outcome of a dynamic, interactive network of beliefs (and that it reciprocally influences them).

It also is unclear just what causes what. Does a trigger–a real-world or imaginal event–activate core beliefs or automatic thoughts? Once set in motion, which causes which? Beck (2011) has little to say about the relationships between automatic thoughts, intermediate beliefs and core beliefs other than core beliefs “activate” automatic thoughts (p. 32) and “underlie” (p. 36) both them and intermediate beliefs. Intermediate beliefs “influence” one's view of the situation or event (p. 35), which “trigger” automatic thoughts (p. 38) (Beck apparently views these different verb formulations as synonymous).

### Belief revision–three and only three fundamental syntactical operations

While CBT provides useful tools that can be used to induce or facilitate belief revision such as cognitive restructuring or exposure/response prevention, the problems with Beck's (2011) formulation (§2.3, above) make clear that it comes up short to explain just how they do so. At best, from a clinical standpoint, they just “soften” a set of dysfunctional beliefs, or point out why individual beliefs are implausible (Beck) or illogical (Ellis). We contend that the process of belief revision in CBT can be better characterized using AGM^12^.

According to AGM, a person's knowledge base *k* comprises a number of individual beliefs, BEL_1_, BEL_2_,… BEL_*n*_, which combine together to form belief sets. AGM provides a set of ecological rules for how beliefs dynamically evolve by examining the interaction effect of *k*_1_'s and *k*_2_'s respective belief sets at equilibrium points *t*_1_ and *t*_2_ during the process of belief revision. The problem AGM is trying to solve is to minimize the set of BEL_new_ ∈ *k*_2_ and the set of BEL_old_ ∉ *k*_1_ *simultaneously*, so as to maximally preserve both *k*_1_'s and *k*_2_'s inductive cores. Unlike *k*_1_, *k*_2_ is less subjectively distressing and leads to more adaptive or normative behavior.

This is interesting and important because it defines the necessary and sufficient conditions for belief revision–what has to happen and that is all that has to happen. It therefore specifies the minimum requirements necessary for successful cognitive restructuring or belief modification following exposure/response prevention. From a clinical standpoint, maybe this is all one can expect, particularly with difficult cases. It can accommodate a diverse belief set, limited only by one's strategies to interpret beliefs, semantically encode them by assigning them substantive propositional content (that “*x*”) and then identify the resulting doxastic commitments, which gives it explanatory power. It deemphasizes the distinction between automatic thoughts, intermediate beliefs and core beliefs. All beliefs are targets for revision at any equilibrium point. This better explains the subjective phenomenological experience of belief revision. It also recognizes there are different related beliefs at *t*_1_, *t*_2_, etc. Some motivate behavioral change, e.g., *k*_1_ = (“If I enact behavioral experiment *y* then *z* will happen”). Others reinforce it, e.g., *k*_2_ following skills acquisition or exposure/response prevention = (“This is how the environment responded”). It is a dynamical system because it changes and evolves in real time. It is non-linear because the “*x*” of BEL(*x*) is idiographic, idiosyncratic and unpredictable.

During belief revision, elements of belief sets are modified or replaced using three (and only three) fundamental syntactical operations, which are expansion (EXP); revision (REV); and contraction (CON). Particular beliefs are the semantics this architecture supports (Fermé and Hansson, 2011).

### Expansion (EXP)

EXP is like adding a new belief without deleting any old ones. EXP (expressed as *k*_1_ + BEL*x*) occurs when one accepts, acknowledges or incorporates a BEL_new_ into *k*_1_. *k*_2_ = (*k*_1_ + BEL_new_): BEL_new_ is added to *k*_1_; no ∃(BEL *x* ∈ *k*_1_) is deleted or removed from *k*_1_; and on conclusion of belief revision, {(BEL_1_… BEL_n_) ∪ BEL_new_} ⊆ *k*_2_, with the caveat it also is the smallest possible set of (*k*_2_ ∪ BEL_new_). Although it might be, BEL_new_ does not necessarily have to be consistent with *k*_1_. Since AGM does not restrict the substantive propositional content “*x*” of BEL_new_ (§1.3, above), it can have either ⊕ or ⊖ valence. If it has ⊕ valence (BEL*x*^⊕^), then it contributes to cognitive restructuring at *t*_2_. If it has ⊖ valence (BEL*x*^⊖^), then either it does not contribute to cognitive restructuring, or may even reinforce *k*_1_.

**Figure 3:**
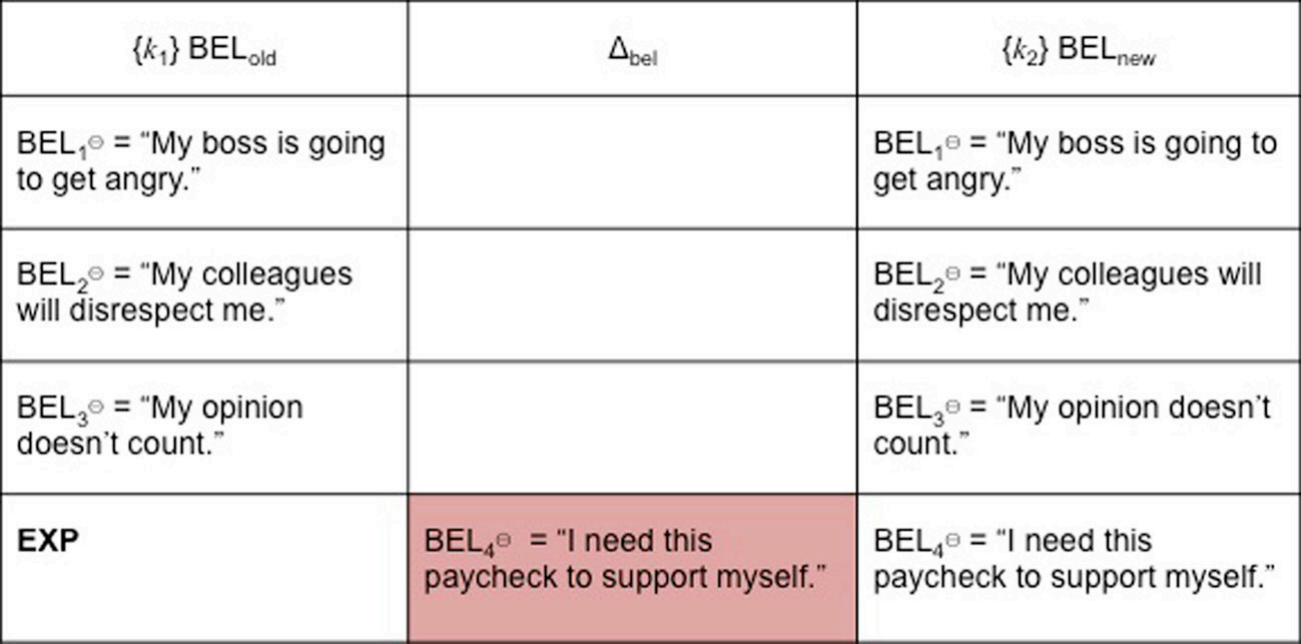
**EXP**.

For this reason, EXP might be confusing for an AGM agent. BEL_old⊖_ remain as elements of her belief set, even as they are joined by BEL_new_, which can either be BEL^⊕^, BEL^⊖^ or ambiguous. To continue with our previous example, the trigger is running late for a meeting at work because one's bus is late. Under such circumstances, one's beliefs might be: BEL_1⊖_ (“My boss is going to get angry”), BEL_2⊖_ (“My colleagues will disrespect me”) and BEL_3⊖_ (“My opinion doesn't count”). One then acquires a new belief BEL_4⊖_ (“I need this paycheck to support myself”). BEL_4⊖_ is not inconsistent with {BEL_1⊖_, BEL_2⊖_, BEL_3⊖_}. For these reasons, we hypothesize that it is unlikely EXP alone will result in successful cognitive restructuring or belief consolidation following exposure/response prevention. Figure 3 depicts this outcome.

### Revision (REV)

REV is like adding a new belief and deleting old, inconsistent ones. As with EXP, REV (expressed as k^*^_1_ BEL*x*) occurs when one accepts a BEL_new_ or admits it to one's *k*_1_ knowledge base. *k*_2_ = (*k*_1_ + BEL_new_): BEL_new_ is added to *k*_1_; on conclusion, {(BEL_1_… BEL_n_) ∪ BEL_new_} ⊆ *k*_2_. The main difference between REV and EXP is that with REV, a BEL_old_ must be *deleted* from *k*_1_ so that *k*_2_ is consistent with *k*_1_.

#### Pragmatic closure

*k* is “logically closed” if it represents *all* of one's beliefs, even though they may be difficult or impossible to specify. Every BEL logically derivable from *k* already ∈ *k*, i.e., *k* includes not only BEL but also all BEL consequences. Stand-alone beliefs sometimes are referred to as “basic beliefs” and consequences as “derived beliefs”–those beliefs one is epistemically committed to hold, even though one might not actively do so (Gabbay et al., 2010). Since *k*_1_ is logically closed in this sense, only *one* anomalous BEL(*x*) is sufficient to create inconsistency; an inconsistent *k*(*x*) sometimes is notated as *k*(*x*)⊥. In this respect, REV incorporates the concept of conformity (§1.6.1, above)^13^.

#### Frame of discernment

To some extent the problem of logical closure is solved by the concept of “frame of discernment.” The domain of all possible beliefs must be truncated in order to engage in practical inference and reason from belief to action. One's frame of discernment is the set of all of the beliefs comprising *k* that are useful to answer, in a practical context, the question of what one believes. It is notated Θ where (BEL ∈ Θ ∈ *k*); we might say one's Θ is “pragmatically closed” in order for one to be able to function effectively in the world. Example: when one adopts the set Θ_1_ = {red, white, yellow} as the frame for the question “What color rose is Bill wearing today?” one formalizes the variable *x* with those possible values. The frame Θ_2_ = {white, blue} might answer the question “What color shirt is Bill wearing today?” The frame for the conjoined question “What color rose and what color shirt is Bill wearing today?” is Θ_1_ × Θ_2_ = {(red, white), (red, blue), (white, white), (white, blue), (yellow, white), (yellow, blue)} (Liu et al., 1991). Frame of discernment narrows down a potentially unwieldy set of beliefs into something more pragmatically serviceable^14^.

**Figure 4:**
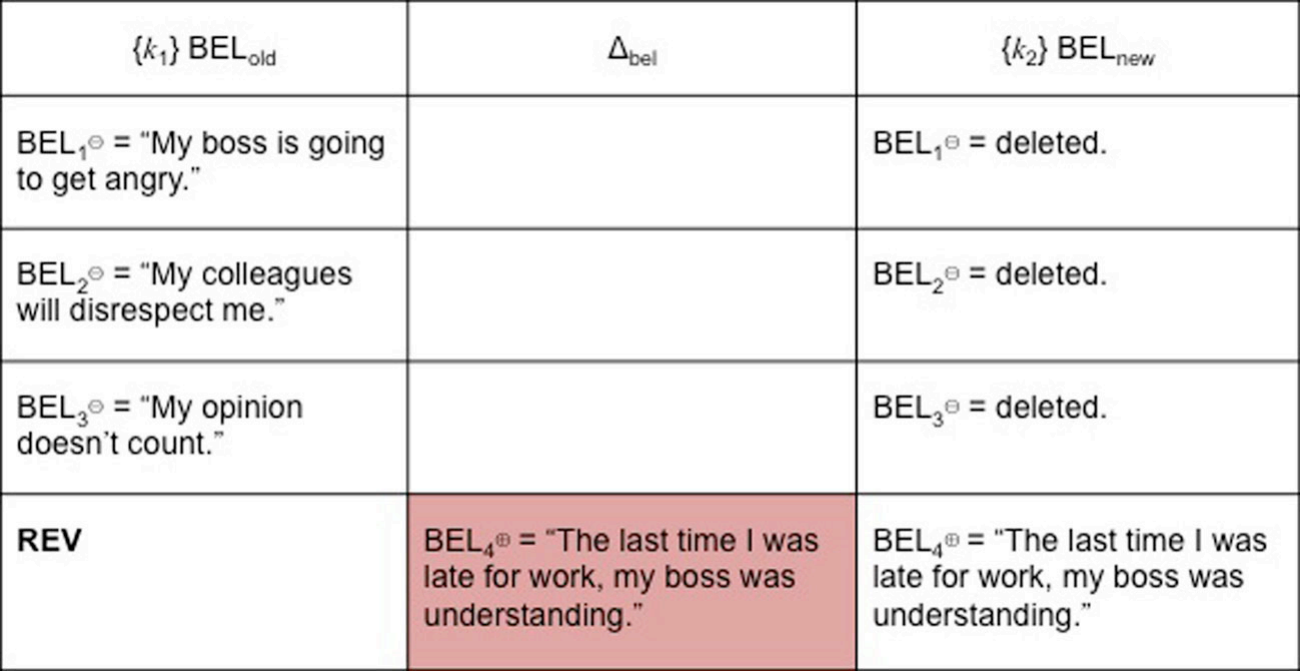
**REV**.

To continue with our earlier example, let's say that at *k*_2_ one has acquired BEL_new⊕_ (“The last time I was late for work, my boss was understanding”). Because it is BEL^⊕^, it is inconsistent with {BEL_1⊖_, BEL_2⊖_, BEL_3⊖_}. The objective of cognitive restructuring or belief consolidation following exposure/response prevention is for *k*_1_ to be *in*consistent with *k*_2_. It follows that BEL_old_ should be BEL^⊖^ and BEL_new_ should be BEL^⊕^, otherwise, there would not be any therapeutic change. Cognitive restructuring is teleological in that it is undertaken with a specific objective in mind, which is belief change and resulting behavior modification. For these reasons, we hypothesize that REV is the paradigm case of successful cognitive restructuring (see Figure 4).

### Contraction (CON)

**Figure 5:**
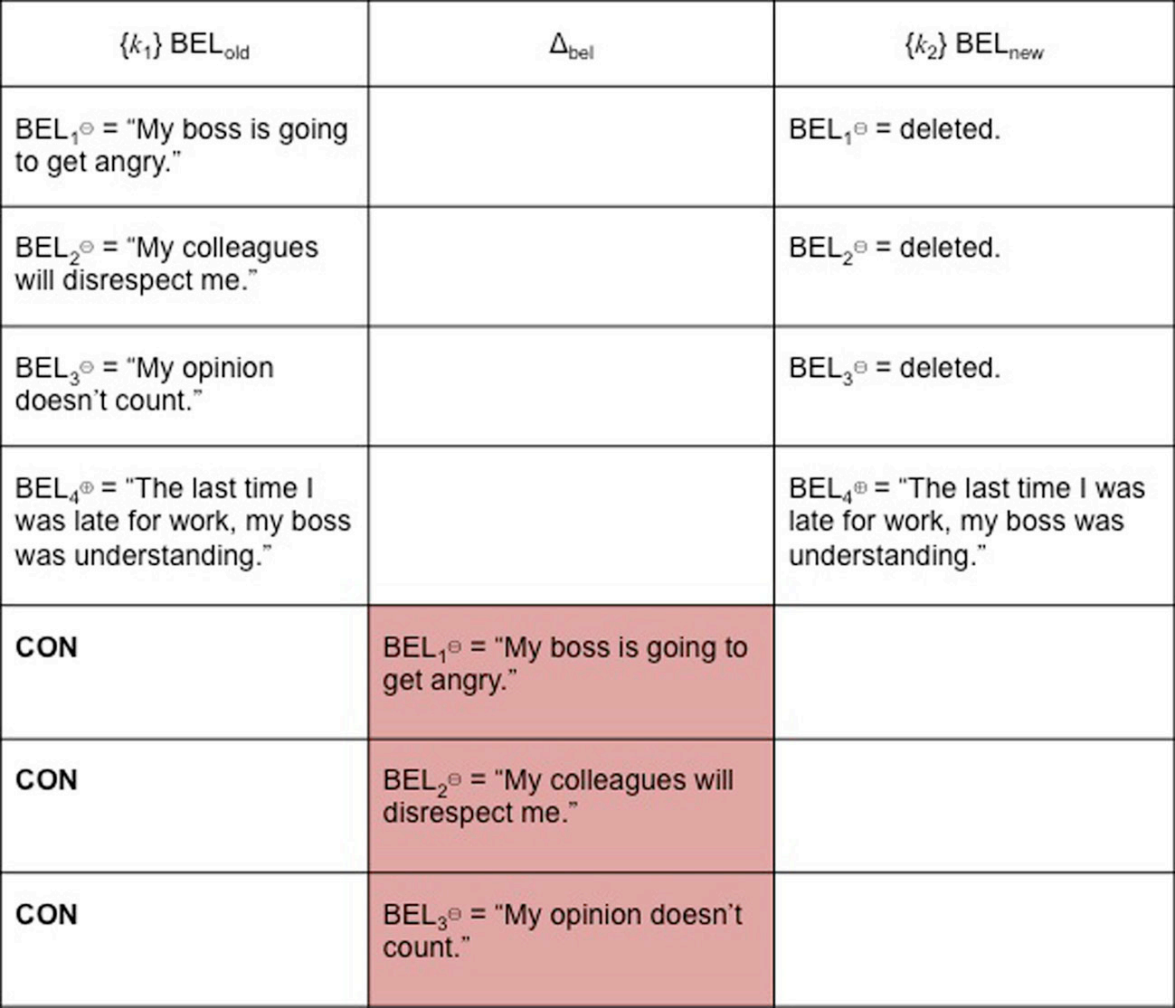
**CON**.

CON is like deleting an old belief without adding any new ones. CON (expressed as *k*_1_ ÷ BEL*x*) is when one rejects a BEL_old_ or deletes it from her knowledge base. *k*_2_ = (*k*_1_ − BEL_old_): *k*_2_ supersedes *k*_1_; *k*_2_ ⊆ (*k*_1_ | *k*_2_ ↛ BEL_old_); but from which no (BEL*x* ∈ *k*_1_) has been unnecessarily deleted. Because a BEL has been deleted from one's *k*_1_ belief set, CON is a process of “epistemic entrenchment.” In rejecting BEL_old_, one also may have to disavow other BEL*x* that imply or are implied by it. Which beliefs should be deleted? From the standpoint of CBT:

1. One should start with those beliefs that violate the requirements of conformity, conditioning and coherence (§1.6.1, §1.6.2, §1.6.3, above). Because of coherence, BEL*x* ∉ *k*_*n*_ trivially is non-entrenched and tautologies are fully entrenched.
2. Next, since an AGM agent strives for minimal change and maximum information value, she should relinquish those beliefs with the least-explanatory power and supporting evidence, because they are less entrenched. The more entrenched beliefs dominate (“≤”) the lesser entrenched beliefs when {(BEL_1_ → BEL_2_) → (BEL_1_ ≤ BEL_2_)} so that *k*_2_ comprises the “inclusion maximal” set (BEL_1_, BEL_2_,… BEL_n_) | (*k*_1_ ↛ BEL_old_) and there is minimal information loss. AGM refers to the beliefs that stay as “remainders.” The remainders comprising *k*_2_ are the maximally-large set of BEL following deletion of BEL_old_ that do not imply any BEL_old_, or their derivatives, remaining in *k*_1_.
3. The exact mix of BEL*x*^⊕^ and BEL*x*^⊖^ selected by CON is determined by the preference function γ (§1.8.1, above), which specifies the minimum set of (BEL*x* ∈ *k*_1_) that ought to be retained in *k*_2_. γ should select *k*(*x*) in order of plausibility; (*k*_2_ γ *k*_1_) represents *k*_2_ as more likely than *k*_1_, given BEL_new_. In other words, γ should select those BEL*x* most likely to result in a more functional (less dysfunctional) *k*_2_. It follows that the most preferred candidates γ should select to delete from *k*_1_ (after steps 3.3.1 and 3.3.2) are BEL^⊖^, such as automatic negative thoughts and their corollary intermediate beliefs and core beliefs, in order to maximize CON's effectiveness. The remainders then will be BEL^⊕^.
4. If γ selects a maximally-consistent set of *k*_1_ that ↛ BEL_old_) to become *k*_2_, then CON is a “partial meet contraction.” If *k*_2_ ends up being populated with only one BEL*x* (unlikely), then CON is a “maxichoice contraction.” If CON selects all of the BEL comprising *k*_1_ (thus *k*_2_ ends up being populated with all of the them), then CON is a “full meet contraction^15^.”
  We hypothesize that CON is the most problematic maneuver for an AGM agent, because its contribution to cognitive restructuring depends on whether it operates on a BEL^⊕^ or a BEL^⊖^. If the BEL that are being deleted are BEL^⊖^, then the remainders will be BEL^⊕^. This corresponds with the intuitive requirement that successful cognitive restructuring should eliminate dysfunctional BEL^⊖^, while leaving BEL^⊕^ alone. On the other hand, it also illustrates a way in which cognitive restructuring might backfire, for example, if one is so committed to a BEL^⊖^ that a BEL^⊕^ is deleted as a consequence. If the belief that is being deleted is a BEL^⊕^, then the remainders all may end up being BEL^⊖^, because they are well-entrenched. An example might be recovery following extinction using a classical conditioning model, which occurs when *k*_1_ ⊆ {(*k*_1_ ÷ BEL_new_) + BEL_old_}. This means that if *k*_1_ was EXP by BEL_old_, but one somehow readopted or reincorporated BEL_old_ into her *k*_1_ belief set, then the effect of cognitive restructuring would be reversed. Or, the BEL set ∈ *k*_2_ could be an ambiguous mixture of both BEL^⊖^ and BEL^⊕^, in which case cognitive restructuring would only be partially successful. Building on our previous examples, Figure 5 illustrates an instance of successful belief revision using CON.

## Integrating AGM into a theory of non-linear dynamical belief revision

We conceptualize belief revision using AGM as an emergent property of a complex, self-organizing system involving huge numbers of neurons broadly distributed throughout different brain regions, including the prefrontal cortex (PFC), Broca's area and Wernicke's area (Cogan et al., 2014). There now has been considerable research imaging regions of the brain activated by BEL(*x*), starting approximately with Greene et al. (2001), continuing through Harris et al. (2008) and d'Acremont et al. (2013). Other studies examine brain regions activated by semantic processing–the words in which beliefs are expressed. Huth et al. (2012) used WordNet (§1.8.2, above) to identify 1705 object and action categories from several hours of nature movies. When they projected them to research participants undergoing fMRI, they were able to map semantic selectivity into smooth gradients covering much of the cortex. Crangle et al. (2013) presented their research participants with 48 spoken-word and visual depictions of sentences about the geography of Europe, half of which were true and half of which were false. They used WordNet and LSA (§1.8.2, above) to extract and classify their propositional content–the *x* in BEL(*x*). The resulting semantic processing was associated with characteristic features of EEG recordings. Costanzo et al. (2013) presented research participants undergoing fMRI with 140 line drawings or pictures of objects (visual stimuli) together with corresponding nouns spoken aloud (auditory stimuli). They found that both converged and were processed in the same regions of the brain during superordinate semantic categorization.

Semantic memory long has been recognized as a fundamental component of human cognition (McRae and Jones, 2013). It is “general knowledge about the world, including concepts, facts and beliefs” and is acquired through experience, thereby “grounding knowledge in distributed representations across brain regions that are involved in perceiving or acting” (Yee et al., 2014, p. 353). Semantic network structure plays a key role in the formulation of ideas and the ways in which they are combined and conceptually associated (Goñi et al., 2011; Marupaka et al., 2012). It accommodates both abstract concepts and concrete ones, the former associated with the medial PFC and the superior temporal sulcus, the latter associated with the bilateral intraparietal sulcus (Wilson-Mendenhall et al., 2013). It represents cognitive information either as specific autobiographical episodes or more general semantic knowledge, each with different subjective experiences (Heisz et al., 2014). Rabinovich et al. (2012b, p. 81) characterize it as a “space of interconnected information items,” where “each item [is a separate] dynamical element” and “the dynamics of thinking (or consciousness) is a flow in a semantic space.”

**Figure 6:**
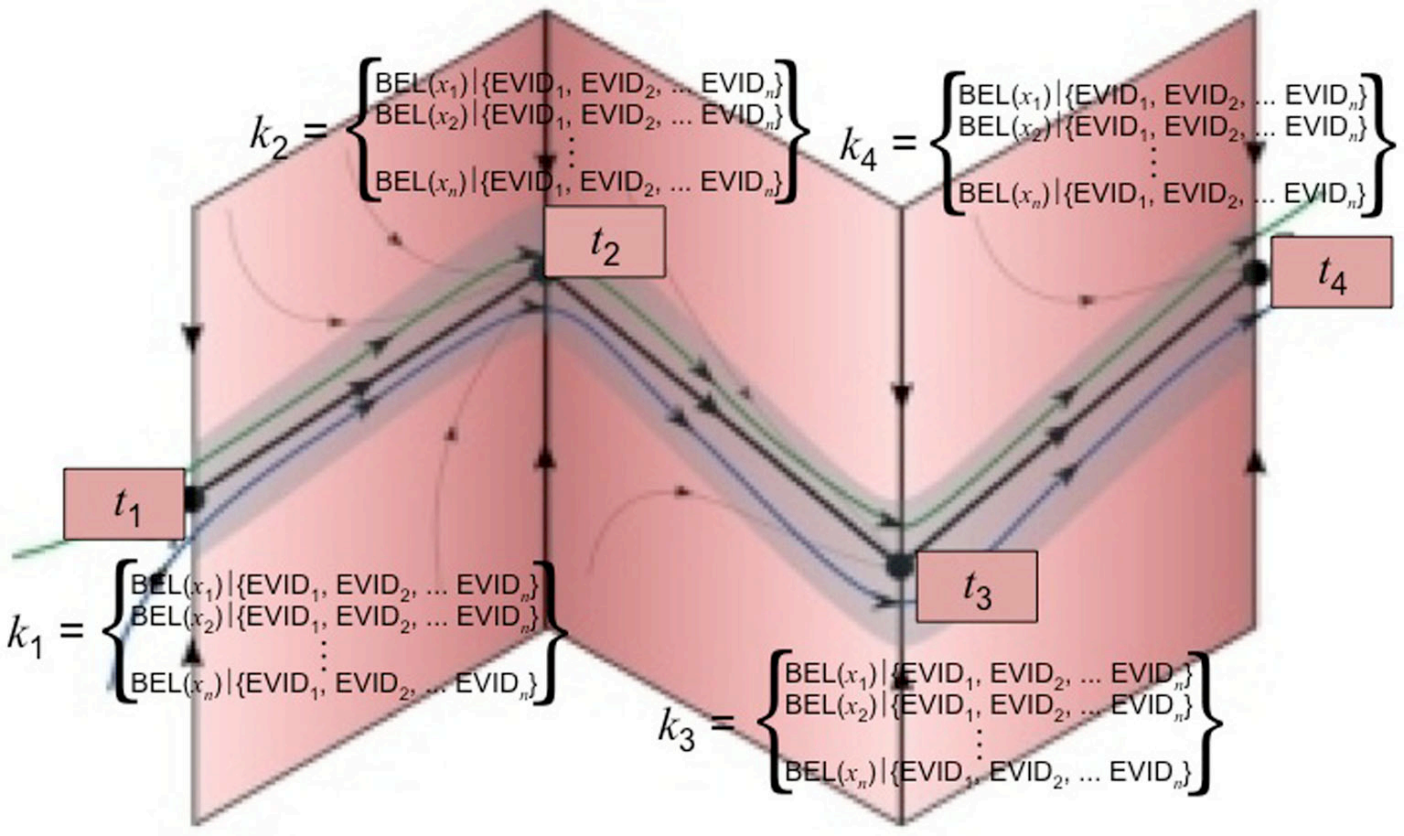
**Hypothesized pathways for belief revision–conceptualization 1.** Adapted from (Rabinovich et al., 2010a). Used with permission.

**Figure 7:**
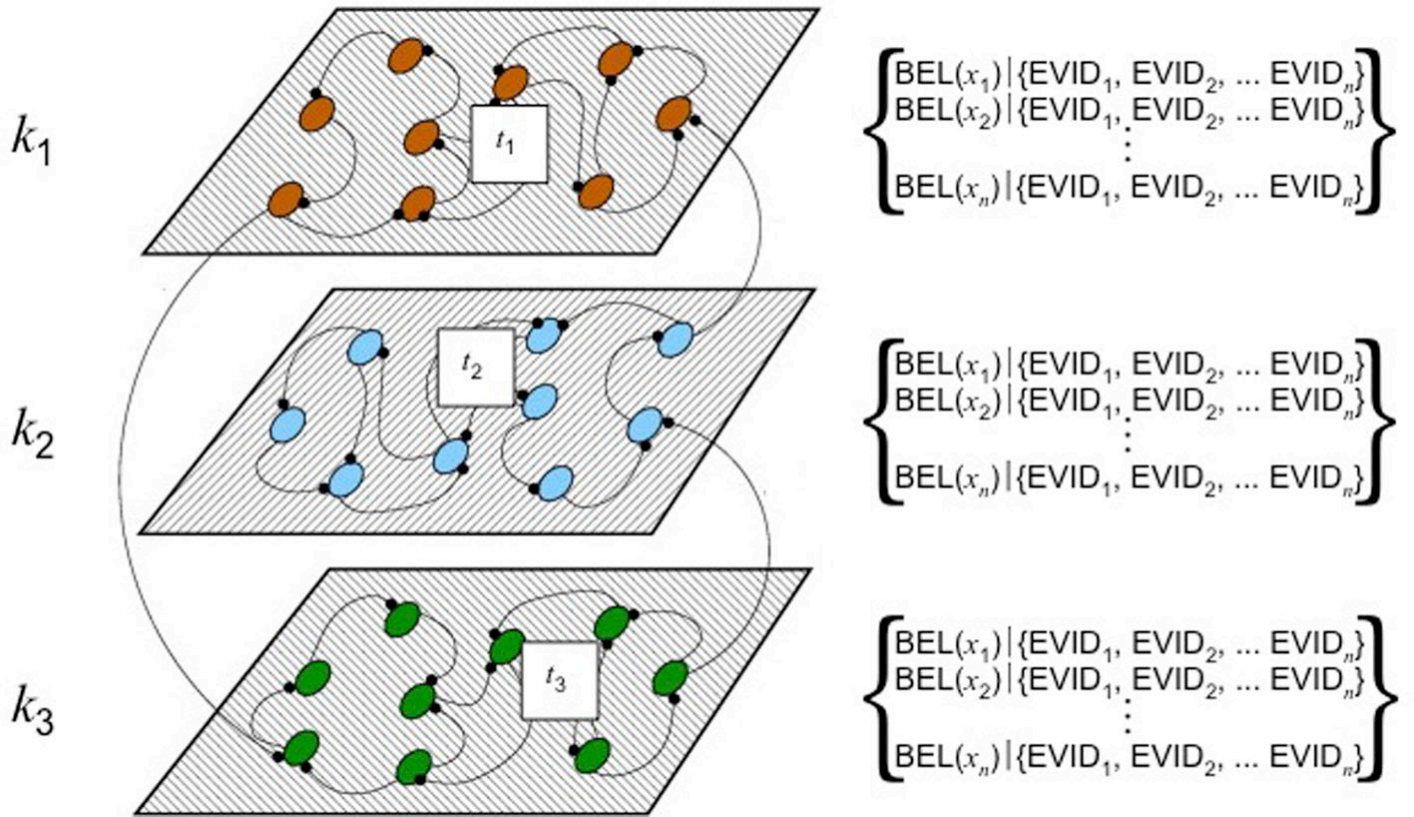
**Hypothesized pathways for belief revision–conceptualization 2.** Adapted from (Rabinovich et al., 2010b). Used with permission.

This body of work supports a conclusion that {BEL | EVID} is not a specific topological location or ontogenetic landscape within the brain. Rather, it is a type of neural activity or pattern of activation that occurs within a comprehensive neural system. When one believes something, one enters into a series of hybrid doxastic/semantic states, which can be functionally represented as a non-linear, dynamical process–a belief revision network occurring in a global workspace–such as that depicted at Figure 6 (while Figure 6 depicts a two-dimensional surface, it should be understood as a multi-dimensional space; Figure 7 depicts an alternative perspective).

It also requires a reconceptualization of the relationship between beliefs and semantics. Unlike an fMRI or EEG recording depicting brain activity, a belief set cannot be described as a geometrical object or in statistical terms. Rather, it is an encoded set of semantic propositions, embodying emergent semantic properties in its very organization (Juarrero, 1999). A belief set creates an internal symbolic mental representation based on one's assessment of its conditions of satisfaction (§1.5, above); one can imagine the conditions of satisfaction being enacted or realized^16^. It interacts with other brain regions responsible for perception, cognition, emotion, language and behavior. They are embedded within a manifold or phase plane together with physiological assets such as blood flow and oxygen. The phase plane is in a constant state of flux, flexibly changing in response to environmental constraints and internal demands (Kelso, 1999). Belief revision is a dynamic pattern of activity occurring within the phase plane.

Some beliefs initially are stored in long-term memory. These most likely are enduring, persistent beliefs about self, others, world and future; background or network beliefs of the sort described at §1.9, above; and core beliefs of the sort described at §2.3.3, above. They are recalled into short-term memory in response to decision points, environmental affordances and outcomes, and other multiple attractors. The network's attractors constitute a “self-organized space with emergent properties that can only be characterized as semantic” because they “embody [word] meaning[s] or sense[s] in the organization of the relationships that constitute the higher-dimensional space” (Juarrero, 1999, p. 167). Initially, the phase plane represents all possible states of the belief-generating and belief-revision systems. It has a large number of degrees of freedom. It is unstable in that small changes to initial conditions–both perceived and imaginal–have the potential to become radically amplified, resulting in any number of different multi-stable belief sets. While the output belief set at *k*_*n*_ depends to some extent on the input belief set at *k*_1_, *k*_*n*_ is asymmetrical and cannot be reliably predicted by *k*_1_. Arguably, it exhibits chaotic dynamics because it would be difficult to specify the individual beliefs comprising the belief set as it evolves into novel and surprising states that are unexpectedly both deterministic and stochastic (non-deterministic) (Nicolis and Prigogine, 1989).

The belief revision system is transient. At *t*_1_, all possible belief trajectories (starting with the system's initial conditions) intersect the phase plane in a structure similar to a Poincaré surface. As it evolves forward in time, it is bombarded with evidence–information derived from its interactions with the environment and subsequent interpretations. It becomes destabilized and undergoes non-equilibrium, dissipative phase transition. Individual beliefs transverse each attractor's basin of attraction and converge into specific belief sets, which consolidate at saddle equilibrium points {*t*_1_, *t*_2_… *t*_*n*_}. They can be conceptualized as a form of Mandelbrot fractal. Broader attractor basins capture or entrain a wider range of beliefs, depending on their strength. Because of the system's chaotic dynamics and each point's turbulent behavior, they resemble strange attractors. Convergence results in heteroclinic binding (Rabinovich et al., 2010b) of different evidentiary data to individual beliefs, which recruit resources and attempt to gain priority using the preference function γ as described at §1.8.1, above. The system bifurcates as new beliefs are formulated based on {BEL | EVID} (§1.1, above), revised conditions of satisfaction (§1.5, above), new evidence/information received as a result of interactions with the environment (§2.2, above), and associated evaluative processes.

Belief revision occurs as belief sets sequentially progress or are deflected from one metastable state to another, forming a heteroclinic channel. The separatices are ridges defining its boundaries. They constrain the flow of resources available to each belief set by modifying the phase plane or the possible trajectories of movements within it. As one belief set begins to dominate, it acquires and sustain coherence, crowding out the semantic space potentially accessible to other beliefs. At some point it reaches critical mass and overcomes an inertial threshold, compelling its migration from *t*_1_ to *t*_*n*_. During this process, the *k*_1_ belief set competes with the *k*_2_ belief set (then *k*_2_ with *k*_3_, etc.) to alter its composition using CON, EXP, or REV, either in response to cognitive restructuring or exposure/response prevention with associated environmental feedback, followed by belief revision.

Since the individual beliefs comprising each belief set displace each other (using CON, EXP, or REV), this is a zero-sum, inhibitory process. The sequence of equilibrium points in the heteroclinic channel form a heteroclinic belief revision network. This process typically remains non-conscious until at *t*_*n*_, when elements of the belief set acquire salience or otherwise are extracted using typical CBT clinical techniques and protocols^17^. The combination of non-linearity and non-equilibrium, context-sensitive constraints initially permits multiple solutions, which have the potential to emerge from and be expressed within a diversified assortment of behaviors (Nicolis and Prigogine, 1989). Numerous beliefs compete in a kind of winnerless competition (Rabinovich et al., 2010a). As it stabilizes, though, the belief revision network appropriates a single behavioral output channel. The behavior semantically satisfies the intentions motivating it (the conditions of satisfaction of the associated belief sets, §1.5, above). Upon its conclusion at *t*_*n*_, the reformulated beliefs comprising the *k*_*n*_ belief set are inserted (or reinserted) back into long-term memory. The behavioral stream transfers to an adjacent nonlinear dynamical system for action. Since emotion regulation also plays an important role in belief revision (Boden and Gross, 2013), associated emotions also are reregulated (§2.1.4, above)^18^.

Cognition and behavior comprise a single autocatalytic unit and it is difficult to assess their respective influences at any *t*_*n*_. Neurocognitive methods do not yet have sufficient precision to discriminate between the two (Morrison and Knowlton, 2012). There are no studies persuasively isolating the cognitive component from the behavioral one. Both require selective deployment of attentional, cognitive and affective resources. Unless belief revision was assessed immediately following cognitive intervention, before enactment of any behavior, it would not be possible to isolate the floor effect of cognitive change and control for reinforcement effects, because cognitive change already would be in the process being incrementally reinforced (for an early and unpersuasive attempt to do so based on the concept of “self-focused attention,” see Wells, 2006). Any kind of change arguably results in a form of behavior. A recent study on the efficacy of mindfulness-based cognitive therapy (Kuyken et al., 2010)–seemingly, the paradigm case of a cognitive intervention–correctly noted that “these interactive mediation effects indicate that treatment changes the nature of the relationship between cognitive reactivity and outcome” (p. 1110).

What we can say is that together, they comprise a heterogeneous, self-organized, complex adaptive system (Juarrero, 1999) (in this sense, realizing Beck's concept of cognition as an information processing system, §2.3.3, above). Both are temporally and contextually embedded, exchanging information and energy with each other depending on the task at hand, the level of one's skills or expertise to accomplish it, and feedback from the environment. Structure and patterns emerge from repeated cycling involving the cooperation of many individual parts (Thelen and Smith, 2000). Although the system initially is out of equilibrium, with high entropy, it self-organizes by assuming a structure allowing it to operate more efficiently (Guastello and Liebovitch, 2009). Repeated behavioral stimulation and learning history facilitate signal transmission between neurons. Neural plasticity promotes Hebbian-type long-term potentiation, which in turn cascades into further hybrid cognitive-behavioral activation and reinforcement, strengthening attractors and facilitating the development of more predictable belief trajectories within the semantic phase plane. “Through repeated activation of a pattern the connections between units that are activated simultaneously become stronger and the whole pattern becomes an attractor.” Thus, even if only partially activated, “the network can complete the pattern by a process of iterative spreading activation” so “the previously learned pattern is recovered in a number of updating cycles in which the activation level of each unit is adjusted according to the activation levels of the other units and the strength of the connections between the units” (Pecher, 2013, p. 359). As a result, conditions of satisfaction (§1.5, above) are revised, together with their corresponding internal symbolic mental representations (§1.1, above). These brain-environmental interactions comprise a negative feedback loop if they increase the incidence of target behavior; a positive one, if it decreases.

From a clinical standpoint, many cognitive interventions (such as mindfulness) are inherently mental and remain thoroughly solipsistic even as they reinforce and are reinforced by new behavior. Many principles of acceptance and commitment therapy (ACT) are cognitively front-loaded, for example, using metaphor as a means of identifying and developing a valued direction and defusing from one's private mental experiences (Hayes et al., 2012). Other examples are motivational interviewing for substance abuse (Miller and Rollnick, 2012); cognitive behavioral analysis system of psychotherapy (CBASP) for depression (McCullough, 2000); and cognitive processing therapy for PTSD (Resick et al., 2002). Behavioral factors, on the other hand, more clearly dominate interventions such as behavioral activation for depression; exposure/response prevention treatment for obsessive-compulsive disorder or attention deficit disorder; and prolonged exposure therapy for PTSD (Foa et al., 2007). With its dual emphases on learning (cognitive) then applying (behavioral) skills, DBT for borderline personality disorder (§2.1.4, above; Linehan, 1993) lies somewhere in the middle.

In some instances behavioral therapy is a more plausible intervention than cognitive therapy, and vice versa. Unquestionably it is possible to train up organisms with little cognitive processing capacity to demonstrate learned behavior. A 700-kg alligator, for example, has a brain that would fit comfortably inside of a teaspoon (Coulson and Herbert, 1981), yet still is capable of learning in the sense of (Squire and Kandel, 1998)^19^. In principle, it would be amenable to behavioral therapy. At some point, though, higher-order propositions must be expressed using natural language or a natural language equivalent^20^. Without it, propositions would neither be true nor false; the concept of truth builds upon veridical experience. Nor would beliefs have conditions of satisfaction (§1.5, above), nor would psychopathological beliefs have none (§1.6, above). Unlike behavior therapy, cognitive therapy depends on semantics. For this reason, as per §2.1.3, above, it is unclear whether persons with thought disorders can benefit from it (compare Grant et al., 2012 with Aggarwal and Basu, 2013; for a current overview, see Bachman and Cannon, 2012; and Jauhar et al., 2014). While of course outcomes lie on a continuum, arguably, it would be ineffective in principle for those toward the far end of the spectrum. If a person remains impervious to environmental feedback–she is unable to develop adaptive cognitions and activate belief revision–we are inclined to say that something is impeding the assimilation of new evidence, or that her information processing systems require recalibration. Functionally, she may be in a concrete operational stage, or otherwise incapable of abstract thought or metacognition. Having a theory of mind–being able to think about thoughts–may be a necessary component of psychological change (Saxe and Young, 2014). One solution from an operant conditioning perspective might be to increase positive reinforcement (R^⊕^) or to titrate down punishment using negative reinforcement (R^⊖^) in order to upregulate the desired behavior, with a view toward mobilizing additional cognitive resources.

**Figure 8:**
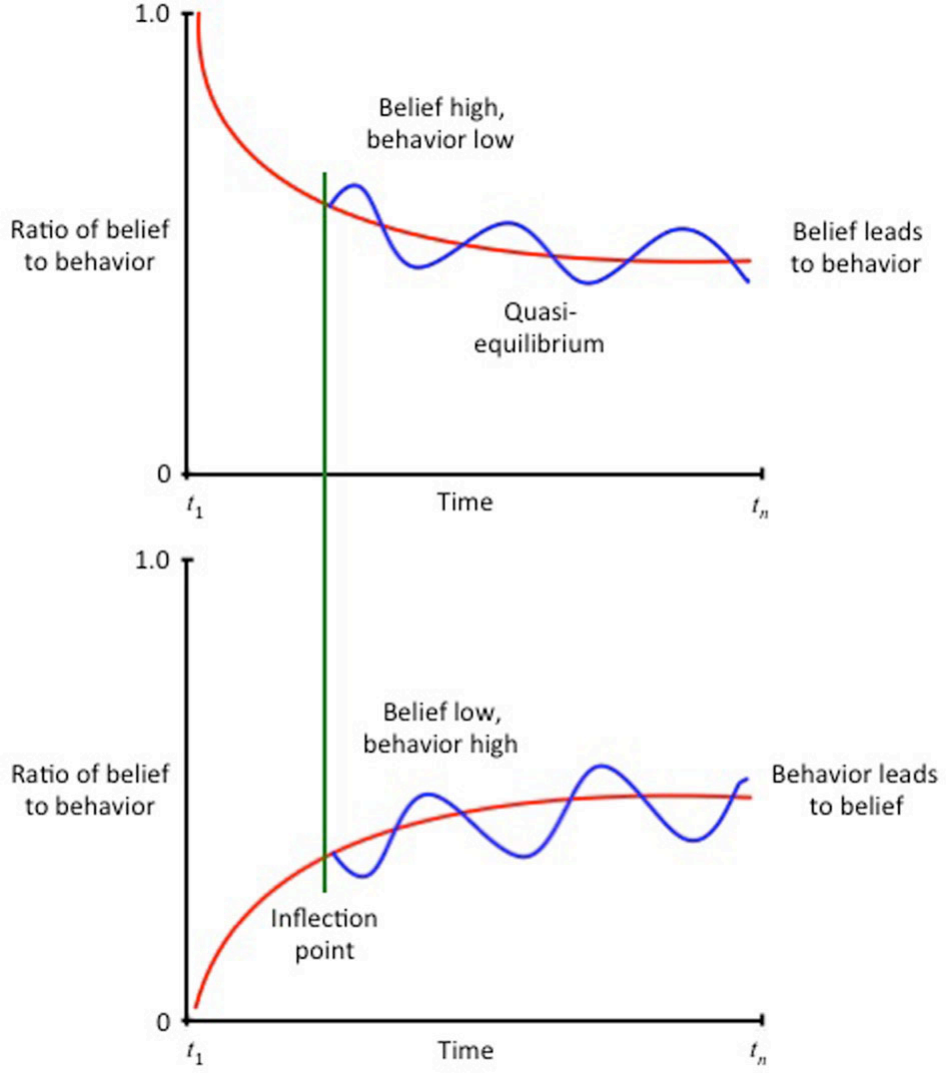
**Transactional relationships between beliefs and behavior–conceptualization 1**.

**Figure 9:**
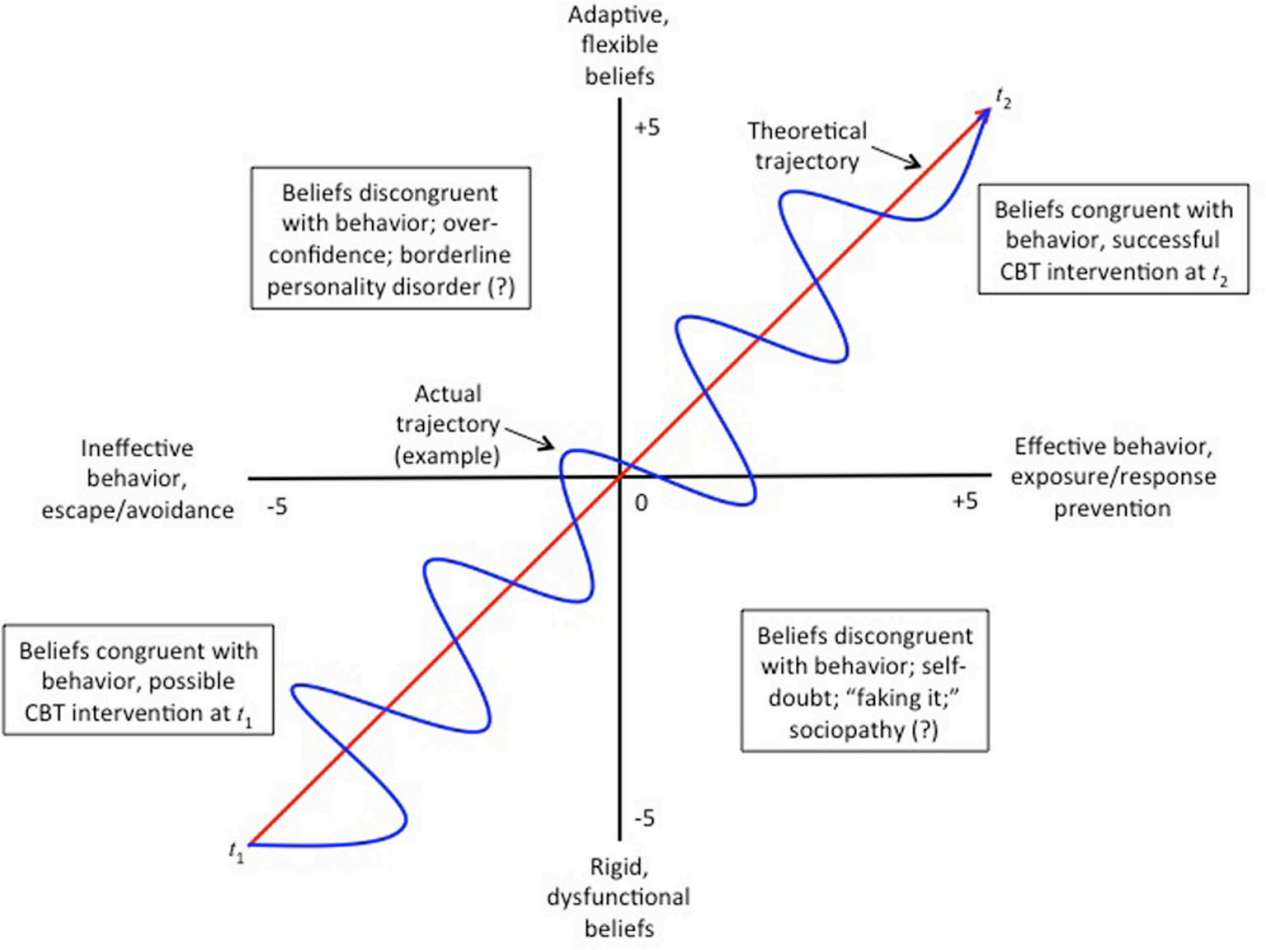
**Transactional relationships between beliefs and behavior–conceptualization 2**.

Most likely cognition and behavior shuttle back and forth quickly depending on the client's perceptions, emotions, language capability, attentional focus, the context in which behavior occurs, the nature of the transaction the client is having with her/his environment, experience/learning history, genetics, neurochemistry, interoceptive sensitivity, memory capacity, heuristics, intuition, vulnerabilities, intentions, skills, values, and a variety of other factors. Their different trajectories oscillate (Schultz and Heimberg, 2008) in what Rabinovich et al. (2010b) would characterize as a heteroclinic channel between metastable states. Because the brain is a complex system with a variety of different inputs and outputs, neither cognition nor behavior can be controlled in isolation (Ruths and Ruths, 2014). From a clinical standpoint, target behavior should progressively and dynamically reduce. As depicted at Figures 8, 9, their relationship is transactional. The exact mix of each depends not only on the type of therapy but also stages in the therapeutic process. For example, the manic phase of bipolar disorder (DSM-5 §296.xx) might be more amenable to cognitive therapy, whereas the depressive phase might be more amenable to behavioral therapy (Leahy, 2005). Daugherty et al. (2009) characterized this as a Liénard oscillator with autonomous forcing. From the standpoint of belief revision semantics, the theme of the substantive propositional content (“*x*”) remains the same, even as the propositional attitude toward it changes, e.g., if the domain is “affection,” then manic = “adorable” whereas depressed = “unlovable.” Conceptually, behavioral reformulation and cognitive reconstruction serially propel it in a dynamic progression from *t*_1_ through *t*_*n*_ as different inhibitory and stimulating paradigms take effect. At some point in this process–an extremely interesting one from the standpoint of cognitive science–their trajectories intersect and one transitions into the other. Both are active ingredients of therapeutic change.

## Conclusion

The ultimate goal of cognitive restructuring or belief consolidation following exposure/response prevention should be thorough overhaul of a meaningful subset of one's entire belief system. Simply inducing doubt is not sufficient. An example of such a paradigm shift might be a prisoner on death row who is exonerated by new DNA evidence, resulting in radical reformation of her knowledge base, or Dostoyevsky's experience in front of a mock firing squad (Bloom, 2005). This is every bit as profound and disruptive as the transition from Ptolemaic astronomy to Copernican astronomy, or from Newtonian physics to Einstein physics, or through the so-called three waves of cognitive behavioral therapy (Hayes, 2004). Thomas Kuhn (1962/2012) labeled these “scientific revolutions”–on an individual level, they might be labeled “personal revolutions.”

In addition to making a case for AGM, one of our main objectives in this review has been to illustrate a point of intersection between cognitive science and clinical psychology, two fields which long have enjoyed an uneasy *rapprochement* (Macleod, 2010). “The study of psychopathology has… become an important facet of the cognitive sciences, and the cognitive sciences have, in turn, exerted an important influence on many regions of psychiatry” (Cratsley and Samuels, 2013, p. 413). One of the characteristics of many cognitive science theories is that while each step of the argument makes sense, when viewed as a complete chain of inferential reasoning, the transition from premises to conclusion may be implausible, in a C.P. Snow (1959/2012)-type sense. Like a salmon swimming upstream, one ends up in a very small pond. Clinical psychology, in turn, depends operationally on protocols that first were devised over a quarter of a century ago. The prospects for *détente* are not as far-fetched as they initially might seem. For example, on April 1, 2014, the Max Planck Society announced a €5 million investment in a new center for computational psychiatry to be based in London and Berlin, with a view toward uncovering relationships between cognition and psychopathology of the sort we hypothesize (Siddique, 2014).

We submit that the best way to think of our initiative is that it is an exercise in translational research. It applies a form of non-linear analysis to the study of complex systems in cognitive science and behavioral analysis. Even though it may not exactly mirror actual, common sense psychological activity, logical reasoning should “clarify, sharpen, systematize the purely semantic-level characterization of the demands on any such implementation, biological or not” (Dennett, 1984/2006, p. 449); to “provide an account of our cognitive architecture–which specifies the basic operations, component parts, and organization of the mind” (Samuels, 2012). It also demonstrates how recent work in experimental cognitive science can be combined with clinical psychology to inform the process of psychological change.

### Conflict of interest statement

The authors declare that the research was conducted in the absence of any commercial or financial relationships that could be construed as a potential conflict of interest.

## Footnotes

^1^Some of the other issues affecting beliefs that are beyond the scope of this review include (for starters): the subjective, phenomenological experience of belief; taxonomies of different types of beliefs; the relationship between beliefs and emotions; the role of memory; subjective probability theory; Bayesian epistemology; Dempster-Shafer theory; theories of reasoning; and rationality. In addition we do not here address objections such as logical omniscience, monotonicity and whether language (and beliefs) can be analyzed using a logical structure, to begin with.

^2^In linguistics the study of how language actually is used is known as deixis (Brisard, 2011). Deixis is an example of how one's environment pragmatically imposes itself on one's beliefs. Although a word's semantic meaning may be fixed, what it actually means can vary with a number of factors, such as person, place and time. All of these are susceptible to ambiguous reference if viewed in isolation. It may not be clear, for example, who is designated by a pronoun. Spatial locutions such as “here” or “there” may designate more than one location, and temporal ones such as “now” and “then” might apply to different times (Corazza, 2011; Hanks, 2011). By constraining the limits of potential communication systems, ambiguity in natural languages actually may be adaptive (Piantadosi et al., 2012; Solé and Seoane, 2014). Deictic reference is a sub-category of indexical reference, which expands these principles to any context-sensitive use. Example: a vague expression with a hidden or latent variable, or one that has a particular meaning unique to a local community (such as “urban slang”), which often is uninterpretable absent specialized knowledge (Braun, 2007).

^3^A related concept is intensionality, later developed by Rudolf Carnap (1947/1988). Intension roughly is the same thing as meaning or sense. It contrasts with extension, which roughly is the same thing as reference. For Carnap, two phrases or sentences have the same extension if they designate the same thing, i.e. they both are true or false with regards to it, so that one can be substituted for the other. Intensional ones fail this test, at least for our actual world. There is, however, a possible world or state-description with different conditions, in which there is substitutability of identity. That possible world could be our actual world at a different point in time, or even the knowledge base of different persons. Beliefs, according to Carnap, are neither extensional or intensional, because one can believe *x* but not *y* or *z* without realizing they all refer to the same thing. Phrases or sentences are “intensionally isomorphic” if in fact this intersubstitutability relationship nonetheless exists.

^4^The Human Connectome Project was established in September, 2010 by the U.S. National Institutes of Health (Vance, 2010). In April, 2013, the U.S. announced its BRAIN Initiative, a $1 billion connectionist-type project. It joined a similar €1 billion venture, the Human Brain Project, announced in January, 2013 by the E.U. (Abbott, 2013; Reardon, 2014). Internet companies such as the Allen Institute for Brain Science (Carey, 2012); Google (Markoff, 2012); and Vicarious (Albergotti, 2014) have similar objectives. Because connectionism results in something akin to a static, point-in-time wiring diagram, it is the opposite of non-linear dynamical psychiatry, see §4. Connectionism has obvious applications to artificial intelligence (AI), beyond the scope of this review to investigate further.

^5^Eliminative materialists such as Churchland et al. necessarily are committed to a theory that psychological disorders are a result of brain malfunction, for example, defective or impaired neurochemistry (Matthews, 2013).

^6^Primarily for this reason, it is not clear that a comprehensive Bayesian approach to belief formulation and revision (for a summary, see Davies and Egan, 2013) is viable.

^7^Other than noting its important function, it is beyond the scope of this review to assess γ's mechanism of action.

^8^Obviously this may be any type of language capable of performing this function.

^9^The logical underpinnings of natural languages is an involved subject, beyond the scope of this review; for recent discussions, see Carruthers (2012) and Scholz (2011). Culbertson and Adger (2014) recently concluded that some grammatical rules (such as placing adjectives closest to the noun they modify) are innate.

^10^Davidson also contends that one must be aware one has a belief in order to hold it to begin with, because if one didn't, then one wouldn't be able to change it, because one wouldn't be able to recognize that the underlying belief was false. This type of metacognitive awareness might be helpful for eliciting beliefs, §1.8.2, above. However, we concur with Laurence and Margolis (2012) that such a requirement overstates the case.

^11^While we spend considerable time analyzing pathways between cognition and behavior (§4), it is beyond the scope of this review to expand our analysis to include emotions and affect. For speculation on this point, see (Afraimovich et al., 2011; Huntsinger and Schnall, 2013); and (Rabinovich et al., 2010a).

^12^Since their original (1985) paper, AGM theory has evolved and undergone significant further developments (Makinson, 2003; Costa and Pedersen, 2011; Gärdenfors, 2011). While there are other theories of belief revision (Fermé and Hansson, 2011), AGM is the one that has acquired the most traction in the literature. The concept of *k*, whether and how BEL represents or stands for a psychological state, all of the AGM postulates and all of the operations potentially performable on *k* have been discussed and challenged extensively. It is beyond the scope of this review to analyze these various permutations.

^13^There are several other possible operations one can perform using REV: “partial meet revision” and “transitively relational partial meet revision.” We do not cover these, here. Logical closure may be unrealistic in a real-world environment, because one might not recognize derived beliefs, even if they are specified. One draws on numerous other beliefs, facts assumptions and knowledge about the world in order to function effectively within it. It is unlikely one ever is in command of all possibly relevant evidence pertaining to a belief or beliefs. It most likely would be impossible to specify fully all of the beliefs comprising one's knowledge base, a project that in effect would require axiomatizing all human knowledge (Dreyfus, 1992; Shanahan, 2009).

^14^A related concept is partition dependence, which is the psychological pattern of how one divides up a set of possible outcomes into particular events. Doing so influences the perceived likelihood those events will occur. Combining events into a common partition lowers their perceived probability. Conversely, unpacking events into separate partitions increases their perceived probability (Sonnemann et al., 2013). For example, apocryphally, Eskimos have numerous words for “snow,” because that phenomenon allegedly is far more prevalent where they live than elsewhere (Martin, 1986). They need a vocabulary with greater subtlety and nuance to describe its various aspects. This in turn increases the probability an event will be interpreted as snow-like, because a set of phenomena (e.g. cold wet stuff falling from the sky) with its associated beliefs (e.g. if you stay out in it too long, you will freeze) has been parsed out into separate partitions. Rabinovich et al. (2014, p. 1) recently characterized this as “chunking”–a dynamical strategy agents use to “perform information processing of long sequences by dividing them in shorter information items” thereby making “more efficient use of short-term memory by breaking up long strings of information.”

^15^There are several other possible operations one can perform using CON, including “transitively relational partial meet contraction.” We do not cover these, here.

^16^Mental images are controversial (for a summary of recent work, see Doumas and Hummel, 2012; Markman, 2012; Reisberg, 2014; and Shea, 2013). We are not committed to a theory that one creates actual, static mental representations in the brain. They are not pictures, rather, “depictive representations interpreted by cognitive processes at play in other systems” (Borst, 2014, p. 84). They have “several levels of complexity, from sparse, atomic concepts to complex, knowledge intensive ones” (Rips et al., 2012, p. 177). An agent's behavior must be flexible in order to respond to her circumstances, and mental representations play an important role in enabling her to do so (Egan, 2012, p. 250). Perception, for example, may be more of a process whereby a perceiver skillfully interacts with her environment. The real world presents way too much information for the perceiver to encapsulate it in an isomorphic mental image. Rather it is like a gigantic external memory, supplying a series of cues, which the perceiver can access as necessary (Noë, 2004). We do not, of course, contend that one literally perceives the words comprising the semantic formulation of one's belief set (in a manner similar to the way the Arnold Schwarzenegger character in the movie Terminator III movie was able to scroll through different belief-action options before selecting a particular alternative).

^17^In this we are in accord with Dehaene (2014, p. 8) and Searle (1992, p. 152) to the effect that “The notion of an unconscious mental state implies accessibility to consciousness. We have no notion of the unconscious except as that which is potentially conscious.” Metaphorically, beliefs are like objects within a multi-dimensional hologram; at any given time we are able to observe only a small portion of them within a potentially vast space-time continuum. Our characterization of the belief-generation and belief-modification process does not implicate any particular theory of action or agency, other than the basic principle that behavior is the action-expression of belief.

^18^Though we disagree with Boden and Gross' naive model of how this works (pp. 591-2), which appears to be the result of reading too much literature on acceptance and commitment therapy (ACT).

^19^This is the double entendre behind the title of B.F. Skinner's famous paper “Superstition in the Pigeon” (1947). Superstition is a form of cognition, whereas pigeons only are capable of learned behavior.

^20^There is no bright-line test for this, either. The meaning of simple propositions can be enacted using language-like behavior, such as Quine's famous example of a speaker using ostension to point to a rabbit, while uttering the word “gavagi” to designate a rabbit-like stage or rabbit-like behavior (Quine, 1964).
